# Sensing of SARS-CoV-2 by pDCs and their subsequent production of IFN-I contribute to macrophage-induced cytokine storm during COVID-19

**DOI:** 10.1126/sciimmunol.add4906

**Published:** 2022-09-09

**Authors:** Paôline Laurent, Chao Yang, André F. Rendeiro, Benjamin E. Nilsson-Payant, Lucia Carrau, Vasuretha Chandar, Yaron Bram, Benjamin R. tenOever, Olivier Elemento, Lionel B. Ivashkiv, Robert E. Schwartz, Franck J. Barrat

**Affiliations:** 1 HSS Research Institute and David Z. Rosensweig Genomics Research Center, Hospital for Special Surgery, New York, NY 10021, USA.; 2 Institute for Computational Biomedicine and Caryl and Israel Englander Institute for Precision Medicine, Weill Cornell Medicine, New York, NY 10021, USA.; 3 Department of Microbiology, Icahn School of Medicine at Mount Sinai, 1468 Madison Ave., New York, NY 10029, USA.; 4 Department of Microbiology, New York University, 430 E 29th Street, New York, NY 10016, USA.; 5 Division of Gastroenterology and Hepatology, Department of Medicine, Weill Cornell Medicine, New York, NY 10065, USA.; 6 WorldQuant Initiative for Quantitative Prediction and Department of Physiology, Biophysics and Systems Biology, Weill Cornell Medicine, New York, NY 10029, USA.; 7 Department of Medicine, Weill Cornell Medical College of Cornell University, New York, NY 10021, USA.; 8 Department of Physiology, Biophysics and Systems Biology, Weill Cornell Medicine, New York, NY 10065, USA.; 9 Department of Microbiology and Immunology, Weill Cornell Medical College of Cornell University, New York, NY 10065, USA.

## Abstract

Lung-infiltrating macrophages create a marked inflammatory milieu in a subset of patients with COVID-19 by producing a cytokine storm, which correlates with increased lethality. However, these macrophages are largely not infected by SARS-CoV-2, so the mechanism underlying their activation in the lung is unclear. Type I interferons (IFN-I) contribute to protecting the host against SARS-CoV-2 but may also have some deleterious effect, and the source of IFN-I in the lungs of infected patients is not well defined. Plasmacytoid dendritic cells (pDCs), a key cell type involved in antiviral responses, can produce IFN-I in response to SARS-CoV-2. We observed the infiltration of pDCs in the lungs of SARS-CoV-2–infected patients, which correlated with strong IFN-I signaling in lung macrophages. In patients with severe COVID-19, lung macrophages expressed a robust inflammatory signature, which correlated with persistent IFN-I signaling at the single-cell level. Hence, we observed the uncoupling in the kinetics of the infiltration of pDCs in the lungs and the associated IFN-I signature, with the cytokine storm in macrophages. We observed that pDCs were the dominant IFN-α–producing cells in response to the virus in the blood, whereas macrophages produced IFN-α only when in physical contact with infected epithelial cells. We also showed that IFN-α produced by pDCs, after the sensing of SARS-CoV-2 by TLR7, mediated changes in macrophages at both transcriptional and epigenetic levels, which favored their hyperactivation by environmental stimuli. Together, these data indicate that the priming of macrophages can result from the response by pDCs to SARS-CoV-2, leading to macrophage activation in patients with severe COVID-19.

## INTRODUCTION

The coronavirus disease 2019 (COVID-19) pandemic is caused by severe acute respiratory syndrome coronavirus 2 (SARS-CoV-2), which has already infected hundreds of millions of people worldwide and is responsible for millions of deaths (1–3). Using single-cell profiling of bronchoalveolar lavage (BAL) fluids taken at different stages of the disease and from the lungs of recently deceased patients with COVID-19 (4–8), we and others have described the presence of a large set of proinflammatory cytokines produced by macrophages (4, 6, 8–13), so-called cytokine storm, although this term remains debated (14–16). These observations highlight a dysregulation of myeloid cells in COVID-19 with an accumulation of inflammatory macrophages that associates with disease severity (4, 12, 17–19). Given that only a small fraction (less than 10%) of lung macrophages are SARS-CoV-2 positive (8), how hyperactivation of macrophages occurs is still unclear, suggesting that these cells must receive additional signals when reaching the lungs.

The role of type I interferon (IFN-I) in protection from viral dissemination of SARS-CoV-2 is well documented, because patients with defects in IFN-I or IFN-III responses (12, 20, 21) or who have autoantibodies to IFN-I cytokines (22–24) are susceptible to SARS-CoV-2 infection and prone to progress to life-threatening COVID-19. Moreover, giving IFN-α in the early stage of COVID-19 is beneficial for infected patients (25). However, the role of IFN-I may be more complex (26, 27). Similarly to what has been observed with SARS-CoV-1 (28) and MERS (Middle East respiratory syndrome) (29), the blockade of IFN-I in humanized mice at the chronic stage of SARS-CoV-2 infection attenuates the inflammatory response by macrophages (30). Moreover, targeting the sustained IFN production in the late phase of SARS-CoV-2 infection in mice by blocking the cGAS-STING pathway reduces severe inflammation in the lung (31), and mice injected with IFN-α have increased lethality when infected with SARS-CoV-2 due to the induction of inflammatory cell death in macrophages (32). Furthermore, mice expressing human angiotensin-converting enzyme 2 (ACE2) infected with SARS-CoV-2 reveal an inflammatory role of IFN-I, leading to immune infiltration by cells such as monocyte-derived macrophages and T cells (33). Hence, mortality after SARS-CoV-1 infection is prevented in IFNAR-deficient mice or by using anti-IFNAR monoclonal antibodies (mAbs), without an increase in viral load (28). These data indicate that IFN-I is beneficial at the early stage of infection with SARS-CoV-1/2 but can also have a nefarious role in later stages of the disease. Understanding the source and kinetics of the IFN-I response to SARS-CoV-2 isthus needed and still unclear.

Plasmacytoid dendritic cells (pDCs) are a key cell type involved in antiviral responses because of their unparalleled ability to secrete IFN-I in response to Toll-like receptor 7 (TLR7) and TLR9 signaling (34, 35). pDCs produce IFN-α in response to SARS-CoV-1 and MERS-CoV (36, 37) and can protect mice in a model of mouse hepatitis virus (38). The depletion of pDCs in the murine model of SARS-CoV-1 protects the mice from lethal lung injury (28). pDCs produce high amounts of IFN-I in response to SARS-CoV-2, with a role for TLR7 (39–43). The number of circulating pDCs decreases in the blood of patients (12) and is inversely correlated with the disease severity (43). In contrast, pDCs infiltrate the lungs of patients with COVID-19, with their abundance evolving with the severity of the disease (4). Together, these data highlight a protective role of pDCs during the course of infection, although whether pDCs contribute to disease is not explored. Here, we observed that pDCs were a dominant producer of IFN-I after the sensing of SARS-CoV-2 by TLR7. We also observed that IFN-I produced by SARS-CoV-2–infected pDCs primed macrophages, leading to an inflammatory response with deleterious consequences for patients.

## RESULTS

### The cytokine storm produced by macrophages is associated with IFN-induced signaling in the lungs of patients with COVID-19

**Fig. 1.**
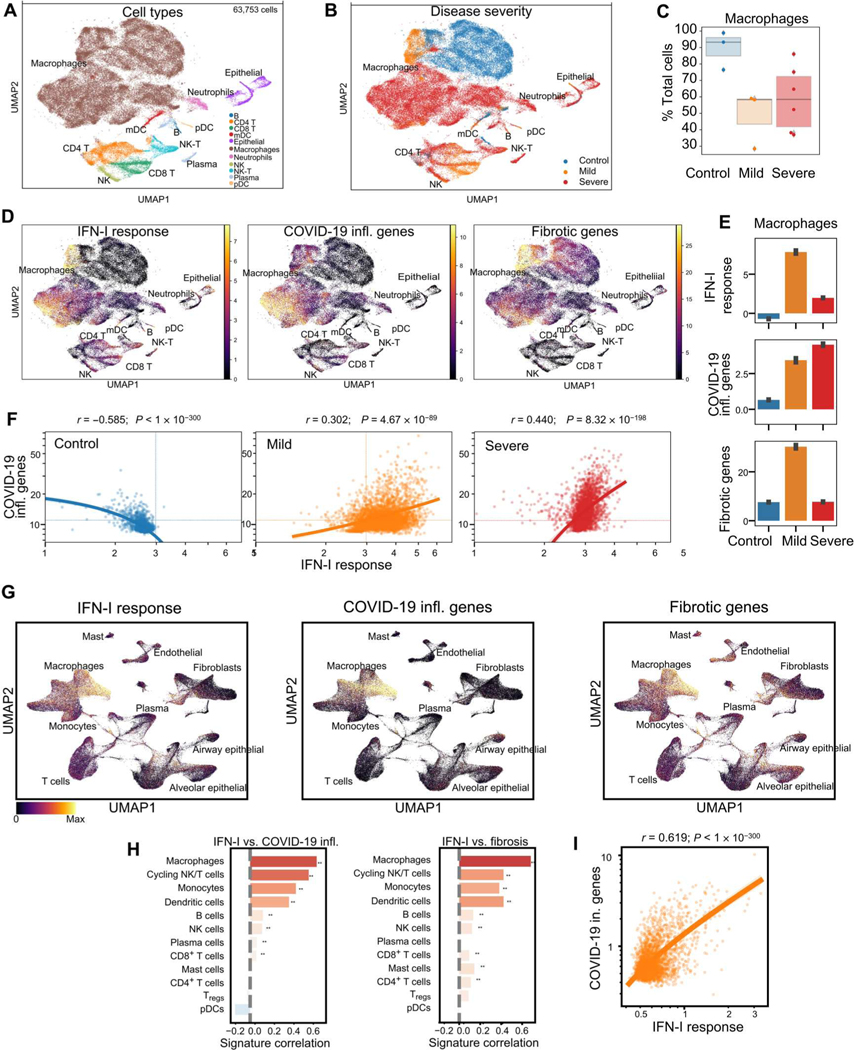
Dynamics of IFN-α and inflammatory responses in macrophages from BAL fluid and lungs of patients with COVID-19. (**A** and **B**) UMAP representation of an scRNA-seq dataset from the BAL of patients with COVID-19 (*n* = 3 mild and *n* = 6 severe) and non–COVID-19 controls (*n* = 4), reanalyzed from DOI:10.1038/s41591-020-0901-9 (4), showing the inferred cell type identities (A) and the disease state of the donor (B). (**C**) Abundance of macrophages depending on disease state in BAL of control patients or patients with mild or severe disease. (**D**) UMAP plot as in (A) in each cell of the BAL showing the intensity of signatures related with IFN-I response, COVID-19 inflammation, and fibrosis. (**E**) Abundance of signatures from (C) for macrophages aggregated by disease state in the BAL from control patients and patients with mild and severe disease. (**F**) Correlation between the signatures of IFN-I response and COVID-19 inflammation across macrophage cells depending on disease state in BAL from control patients (left) and patients with mild (orange) or severe disease (red). The dashed lines mark the mean value of the signatures across all cells. (**G**) UMAP representation of all cells in the snRNA-seq dataset, reanalyzed from DOI:10.1038/s41586-021-03569-1, colored by the inferred activity of signatures related to IFN-I response, COVID-19 inflammation, or fibrosis from lung biopsies. (**H**) Correlation between signature values across single cells of the same cell type in lung biopsy from patients with COVID-19. Significance of Pearson correlation is indicated by asterisks (**) when *P* < 0.01, with *P* values adjusted with the Benjamini-Hochberg FDR method. (**I**) Scatterplot of the inferred activity of IFN-α and inflammatory signatures in macrophages from lung biopsies of patients with COVID-19. Statistics indicate the effect size and significance of Pearson correlation. The line indicates the trend and 95th confidence interval of the data. Note the logarithmic scale of both axes. T_regs_, regulatory T cells; NK, natural killer.

We first analyzed lung samples from patients with terminal COVID-19 (6, 8) and cells from BAL of patients with mild or severe forms of COVID-19 (4). In the BAL samples, we observed a large population of macrophages compared with other cell subsets (Fig. 1, A and B, and fig. S1, A and B), which segregated by disease status (Fig. 1B) but with comparable abundance between patients with mild or severe disease (Fig. 1C). In macrophages and myeloid dendritic cells (mDCs) from patients with mild disease, we observed a strong IFN-I response signature expression (Fig. 1, D and E; fig. S1C; and table S1), whereas the IFN-I response was reduced in macrophages from patients with severe COVID-19 (Fig. 1E and fig. S1C). Of note, the expression of fibrotic genes followed the same pattern as that of the IFN-I–inducible genes, with strong induction in patients with mild disease and reduction in patients with severe disease (Fig. 1, D and E; fig. S1C; and table S1). In contrast, an inflammatory gene signature associated with COVID-19 was very pronounced and sustained only in macrophages of patients with severe disease (Fig. 1, D and E; fig. S1C; and table S1). We analyzed the relationship between IFN-I and inflammatory responses in macrophages at the single-cell level and found that the two programs negatively correlated in controls but positively correlated in patients with COVID-19. Of note, macrophages from patients with severe disease showed the highest correlation (Fig. 1F), because the response in patients with mild disease was more dominated by the IFN-I response (Fig. 1F). This was confirmed by an orthogonal approach using differential gene expression between the three populations of subjects and gene set enrichment. The enriched terms reinforced our conclusion that IFN-I was the main driver of the disease (fig. S2, A to C).

Next, we analyzed the transcriptional profile of total lung cells obtained from recently deceased patients with severe COVID-19 (6, 8) for the presence of genes associated with IFN-I response, inflammation, or fibrosis (Fig. 1G and table S1). Similar to our findings in the BAL data, we observed in the macrophage cluster the presence of a strong IFN-I response and higher expression of genes associated with inflammation and fibrosis (Fig. 1G). These signatures were present in other subsets as well but to a lesser degree than in macrophages (Fig. 1G) and with higher expression in cells from infected patients as compared with control (fig. S3A and table S2), suggesting disease-dependent induction of expression. Of note, there was a strong correlation between the IFN-I response and the presence of profibrotic signals in macrophages (Fig. 1H), which highlights the potential link between IFN-I and the promotion of fibrosis, something that has been suggested in other fibrotic diseases (44). These correlations were present in more than one cellular subset, including other myeloid cells such as DCs or monocytes, but were the highest in the macrophage cluster (Fig. 1H). Similarly to our BAL data, macrophages in lungs had highly significant positive correlation between the presence of IFN-induced signaling and the expression of proinflammatory cytokines in macrophages (Fig. 1I). Hence, these data demonstrate that the inflammatory response by macrophages in the lungs of patients with COVID-19 is tightly associated with the presence of an IFN-I signature.

The presence of an IFN-I response could also be linked to other pathways, such as what we observed with fibrotic genes, and we investigated coagulation cascades and complement activation, which have been reported in patients with COVID-19 (45–47). We observed the presence of coagulation pathways in the lungs at the single-cell level (fig. S4A), with enrichment mostly in nonimmune cell types, such as smooth muscle, fibroblasts, and epithelial cells, whereas complement pathways were also pronounced in immune cells, in particular, macrophages (fig. S4B). Hence, correlation analysis highlighted the relationship between IFN-I response and both coagulation and complement pathways in these cell types (fig. S4C). When we reanalyzed the single-cell BAL fluid from control, mild, and severe COVID patients, we also saw enrichment for an inflammation signature in several cell types but only see enrichment in the macrophage population (fig. S5, A and B). Pairwise correlation between IFN-I, complement, and coagulation pathways across single cells for macrophages was evaluated and revealed a relationship between IFN-I and coagulation. When the relationship between IFN and inflammation pathways was compared for all cell types, the enrichment for macrophages became even more clear (fig. S5, C and D). These data suggest the involvement of the coagulation and complement pathways in relation to IFN-I, in particular, in the macrophage subsets. Overall, these data demonstrate an intimate relationship between the presence of an IFN-I signature and key factors associated with the inflammatory status of lung macrophages, including a set of not only proinflammatory genes but also profibrotic, coagulation, and complement pathways.

### The infiltration of pDCs in the lungs of SARS-CoV-2–infected patients coincides with the peak of IFN-I response

**Fig. 2.**
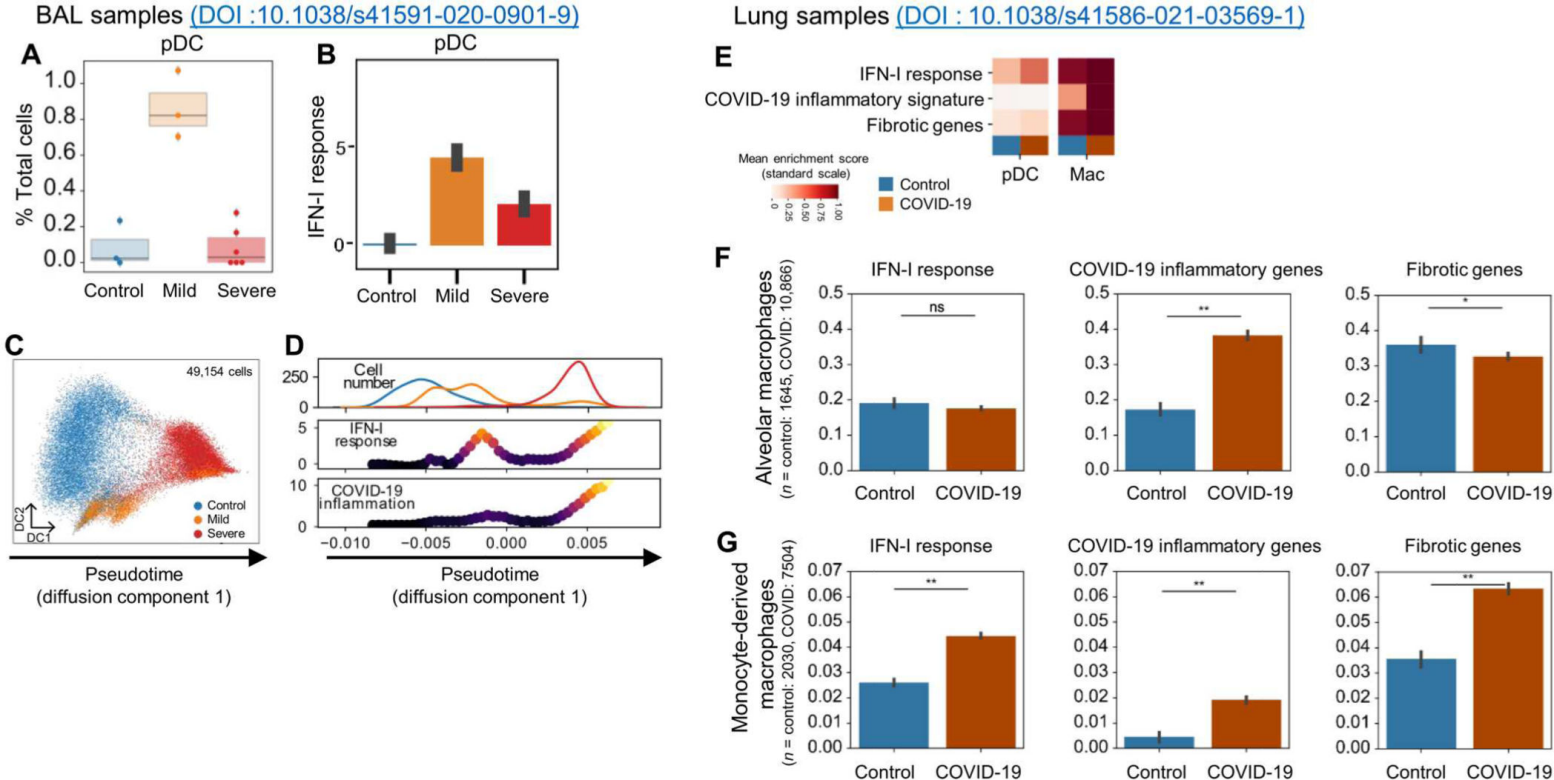
IFN-I response associated with pDCs precedes inflammatory response in macrophages from BAL fluids and lungs of patients with COVID-19. (**A**) Abundance of pDCs depending on disease state in BAL from DOI:10.1038/s41591-020-0901-9. (**B**) Abundance of IFN-I response for pDCs aggregated by disease state in BAL. (**C**) Inference of a pseudotime axis using diffusion maps for macrophage cells only from the BAL of healthy donors (HDs) and patients with mild or severe COVID-19. (**D**) Distribution of macrophage cells (top), IFN-I response signature (middle), and COVID-19 inflammatory signature (bottom) across the pseudotime axis (C) in BAL from HDs and patients with mild or severe COVID-19. (**E**) Heatmap of mean inferred signature activity for pDCs and macrophages from lung biopsies reanalyzed from DOI:10.1038/s41586-021-03569-1, dependent on disease status. Values were min-max scaled per signature to enable comparison. For the signatures, only windows with at least 100 cells are displayed. (**F** and **G**) IFN-I response, COVID-19 inflammatory genes, and fibrotic genes in both alveolar (F) and monocyte-derived macrophages (G) from control and COVID-19 patient lungs. Statistical significance was evaluated with a Mann-Whitney *U* test and *P* values adjusted with the Benjamini-Hochberg FDR method. *P < 0.05 and **P < 0.01. ns, not significant.

To understand the dynamics of the IFN-I response in the lungs of patients, we analyzed the BAL dataset and observed the infiltration of the BAL by pDCs in patients with mild disease (Fig. 2A). The number of pDCs was significantly reduced in patients with severe COVID-19 (Fig. 2A), consistent with the observation by Liao *et al.* (4). These pDCs expressed IFN-I–regulated genes (Fig. 2B), suggesting an activated phenotype, although we could not detect transcripts of IFN-I. These data indicated that in the lungs of SARS-CoV-2–infected individuals with mild disease, up to 1% of the BAL cells were activated pDCs.

We thus hypothesized that the heterogeneous information contained in the single-cell RNA sequencing (scRNA-seq) data can be used to infer a pseudo-temporal continuum of the molecular response to SARS-CoV-2 infection in macrophages. Patients at different stages of the disease could give an indication of the progression of the disease in SARS-CoV-2–infected patients. Using diffusion maps on the transcriptome data of macrophages, we inferred a joint representation and used as the first dimension the dynamics of disease progression (Fig. 2C). Along this pseudotime axis, we observed a high concentration of cells from control patients with low IFN-I and inflammation followed by cells from mild patients with a peak of IFN-I signature, and last, macrophages predominantly from severe patients with high IFN-I and inflammation signatures (Fig. 2D). This is similar to what has been described during influenza virus (Flu) infection, where macrophages respond to IFNs, are influenced by products of Flu infection, and also are a major producer of other cytokines, which can lead to severe Flu (48–50). This unsupervised, dynamic view of the macrophage response reinforces the idea of sequential stages of activation of the macrophages during COVID-19, associated with the early induction of IFN-I at the peak of pDC infiltration (Fig. 2A), which primes macrophages for hyperinflammatory activation in a subset of patients who develop severe disease (4).

The cellular composition and activation status of lung cells in recently deceased patients after SARS-CoV-2 infection have been described (6, 8), but the detailed contribution of pDCs or the role of IFN-I in the disease pathogenesis is unclear. By conducting subcluster analysis in the DC subpopulation in these total lung samples, we identified pDCs (fig. S3, B to G, and table S3), and in comparison with control lungs, the number of pDCs was significantly reduced (fig. S3, H and I, and table S4), although the remaining lung-infiltrating pDCs expressed genes of the IFN pathways as a sign of their activation status in the lungs of the patients (Fig. 2E and fig. S3, J and K). Next, we investigated subsets of macrophages and observed that although both resident alveolar macrophages and infiltrating monocyte-derived macrophages expressed a COVID-19–related inflammatory response, the IFN-I response and fibrotic response were mostly restricted to the monocyte-derived macrophage subset (Fig. 2, F and G). These data indicate that during the course of the disease, pDCs can infiltrate the lung of an infected patient and their presence correlated with a strong IFN-I response in lung macrophages.

### pDCs sense SARS-CoV-2 via TLR7 and are the dominant IFN-I– and IFN-III–producing cells in PBMCs in response to the virus

**Fig. 3.**
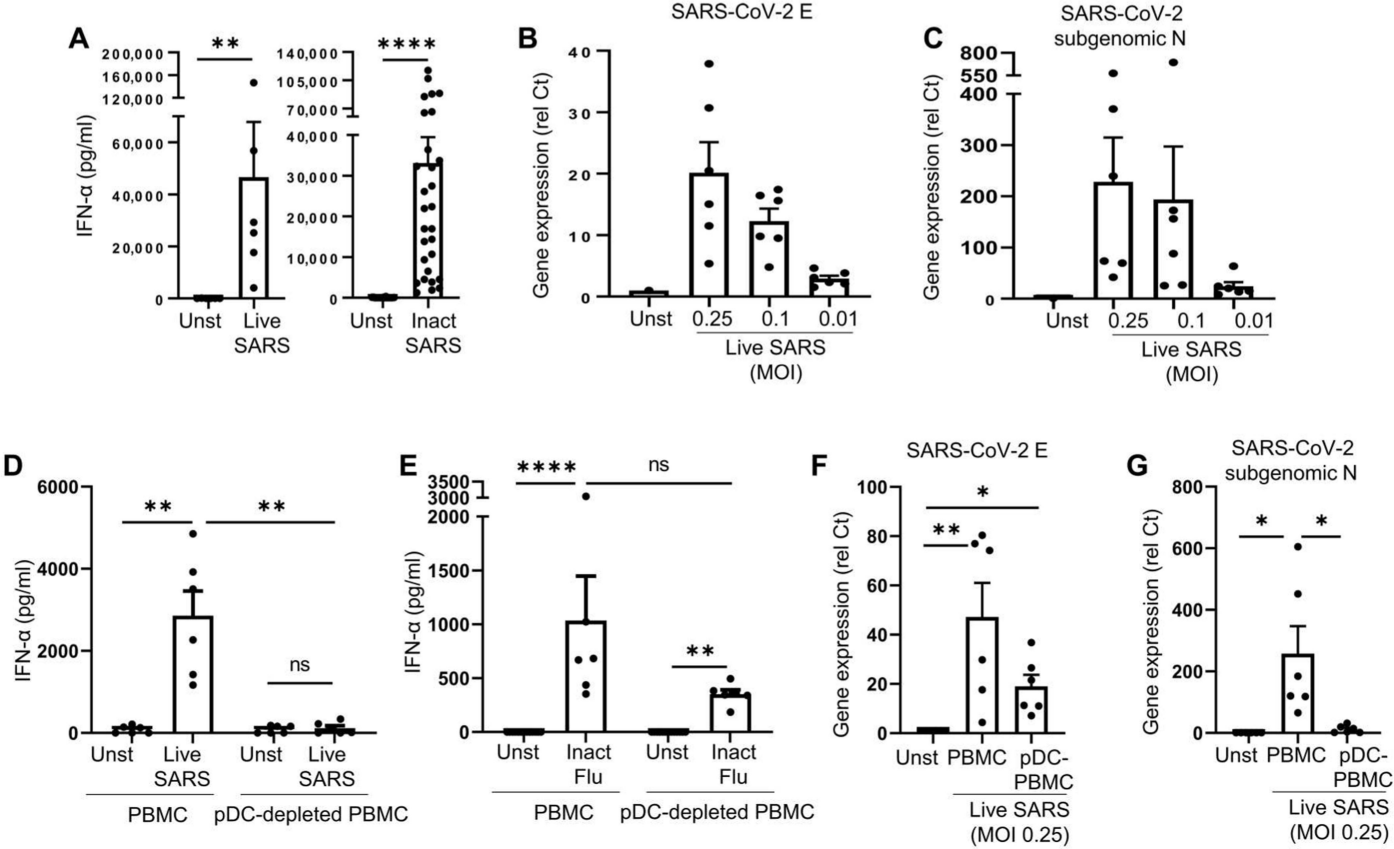
pDCs sense SARS-CoV-2 and are the main producers of IFN-I among PBMCs. (**A**) pDCs purified from PBMCs of HDs were cultured for 24 hours alone [unstimulated (Unst)] or with either live SARS-CoV-2 (*n* = 6) or inactivated (Inact) SARS-CoV-2 (*n* = 29) at an MOI of 0.25. Production of IFN-α was quantified by ELISA. (**B** and **C**) pDCs purified from PBMCs of HDs (*n* = 6) were cultured for 24 hours with live SARS-CoV-2 at an MOI of 0.25, 0.1, or 0.01. Gene expression of SARS-CoV-2 protein E (B) and N (C) was quantified by qPCR (**D** and **E**) or inactivated influenza virus (VR-95, Flu) at an MOI of 2. Production of IFN-α was quantified by ELISA. (**F** and **G**) Total PBMCs or pDC-depleted PBMCs from HDs (*n* = 6) were cultured for 24 hours alone (Unst) or with live SARS-CoV-2 at an MOI of 0.25. Gene expression of SARS-CoV-2 protein E (F) and N (G) was quantified by qPCR. All results are represented as means ± SEM. Statistical significance was evaluated using a Friedman test with Dunn’s multiple comparisons posttest or a Mann-Whitney test. *P < 0.05, **P < 0.01, and ****P < 0.0001.

Although IFN-I is critical for the clinical response to SARS-CoV-2 infection, the cellular source of IFN-I is not well defined. Purified pDCs had a robust IFN-α response to both live and ultraviolet-inactivated SARS-CoV-2 (Fig. 3A and fig. S6, A and B), consistent with earlier findings (40–42), and we observed that the virus was able to efficiently infect the pDCs (Fig. 3B) and replicate in pDCs (Fig. 3C). We then evaluated the relative contribution of pDCs to the overall IFN-I response to SARS-CoV-2 by peripheral blood mononuclear cells (PBMCs). Although total PBMCs could produce significant amounts of IFN-α when incubated with either live or inactivated virus, the production of IFN-α in response to live or inactivated SARS-CoV-2 by PBMCs depleted of the pDCs (pDC-depleted PBMCs; prepared by removing BDCA4-positive cells from PBMCs using microbeads) was negligible (Fig. 3D and fig. S6, C to E). This was in contrast to similar conditions using Flu, where the depletion of pDCs only partially reduced the overall IFN-α response (Fig. 3E and fig. S6F). Consistent with these data, the amount of replicating SARS-CoV-2 in pDC-depleted PBMCs was also negligible (Fig. 3, F and G). These data demonstrated that, in contrast to Flu, where not only pDCs but also other cells could produce IFN-α, pDCs are the dominant producers of IFN-α in PBMCs in response to SARS-CoV-2.

**Fig. 4.**
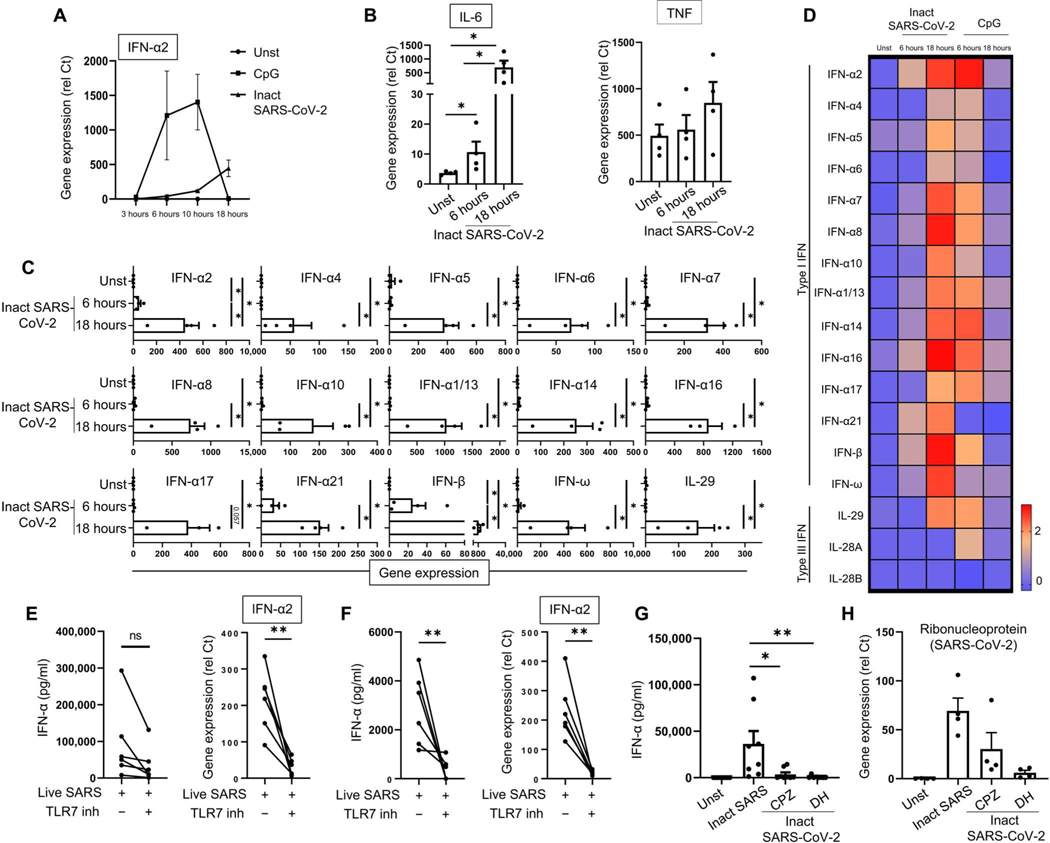
SARS-CoV-2–activated pDCs produce IFN-I and IFN-III via a TLR7-dependent pathway. (**A**) pDCs purified from PBMCs of HDs were cultured in medium alone [unstimulated (Unst)] or with either the TLR9 ligand CpG C274 (0.5 μM) or the inactivated (Inact) SARS-CoV-2 (MOI of 0.25) for 3, 6, 10, and 18 hours (*n* = 4). IFN-α2 expression levels were quantified by qPCR. (**B** to **D**) pDCs purified from PBMCs of HDs (*n* = 4) were incubated for 6 and 18 hours either alone, with SARS-CoV-2 (MOI of 0.25), or with CpG C274 (0.5 μM). Expression of IL-6 and TNF (B) and IFN-I and IFN-III (C) was quantified by qPCR (rel Ct). (D) Heatmap was generated with the log of the mean of each gene. (**E** and **F**) Purified pDCs (E) and PBMCs (F) from HDs (*n* = 6) were cultured for 24 hours with medium only or with live SARS-CoV-2 at an MOI of 0.25 alone or with the TLR7 inhibitor IRS661 (2 μM). Production and gene expression of IFN-α were quantified by ELISA and qPCR, respectively. (**G** and **H**) pDCs purified from PBMCs of HDs (*n* = 4 to 8) were cultured for 24 hours with inactivated SARS-CoV-2 either alone or in the presence of clathrin inhibitor CPZ (30 μM) or dynamin inhibitor DH (100 μM). Production of IFN-α was quantified by ELISA (G), and SARS-CoV-2 ribonucleoprotein was quantified by qPCR (H). All results are represented as means ± SEM. Statistical significance was evaluated using a Friedman test with Dunn’s multiple comparisons posttest or a Mann-Whitney test. *P < 0.05 and **P < 0.01.

To better characterize the response by pDCs to SARS-CoV-2, we studied the kinetics of pDC activation by SARS-CoV-2. pDCs were incubated with inactivated SARS-CoV-2 or CpG, as a positive control, for 3, 6, 10, and 18 hours. Inactivated SARS-CoV-2 induced IFN-α and interleukin-6 (IL-6) and showed a progressive curve, which was higher at 18 hours than at the 6- to 10-hour time points, as for the TLR9 agonist CpG (Fig. 4, A and B). However, the nature of the response by pDCs to SARS-CoV-2 was similar to what is known for TLR7/9 signaling (51), with the induction of all subtypes of IFN-I (Fig. 4, C and D, and fig. S7A) and of a series of chemokines that may contribute to the migration of immune cells into the lungs of patients (fig. S7, B and C). The difference in the kinetics of the response by pDCs between inactivated SARS-CoV-2 and CpG is likely due to the delay in entry of the virus (fig. S7D).

Because SARS-CoV-2 is a single-stranded RNA virus, we explored the mechanism of activation of the pDCs by nucleic acid–sensing pathways. First, we excluded a contribution of ACE2, because these cells have little to no expression of ACE2 (42), and adding an ACE2 inhibitor had no effect on the IFN-α production by SARS-CoV-2–infected pDCs (fig. S8A). In contrast, blocking TLR7 (52) or phosphatidylinositol 3-kinase δ (PI3Kδ), which is key to TLR7-induced IFN-α in pDCs (53), led to inhibition of the IFN-α response (Fig. 4E and fig. S8, A and B). Consistent with our observation that pDCs were the dominant producer of IFN-α in the blood, we observed a complete inhibition of IFN-α production by the TLR7 inhibitor in PBMCs as well (Fig. 4F). Although it is documented that pDCs sense Flu via TLR7 (52), this is different when using Flu, because the inhibition of TLR7 in PBMCs only partially reduced the IFN-α response to Flu (fig. S8C) and had no effect when using pDC-depleted PBMCs (fig. S8D). This finding is consistent with the observation that pDCs from TLR7-deficient patients have a poor response to SARS-CoV-2 (41), although the loss of the overall response to SARS-CoV-2 by pDCs isolated from the TLR7-deficient patients was not complete (41). This may be due to redundancy developed by these cells with germline mutations of TLR7 to virus sensing, as was observed with IRAK4-deficient patients [see (54)]. It is also likely that other cell types that bear TLR7 may be involved in the response to SARS-CoV-2, in particular, in the tissue environment. Nucleic acid–sensing TLRs are located in endosomal compartments (34, 35, 55), and we observed that the entry in pDCs of inactivated SARS-CoV-2 and the subsequent induction of IFN-α required clathrin-mediated endocytosis (Fig. 4, G and H, and fig. S8E). These data thus demonstrated that SARS-CoV-2 could enter pDCs using clathrin-mediated entry and were sensed by TLR7, which signals pDCs to trigger IFN-I production.

### Lung macrophages are not directly infected by SARS-CoV-2 but can uptake SARS-CoV-2 by phagocytosis of infected epithelial cells

**Fig. 5.**
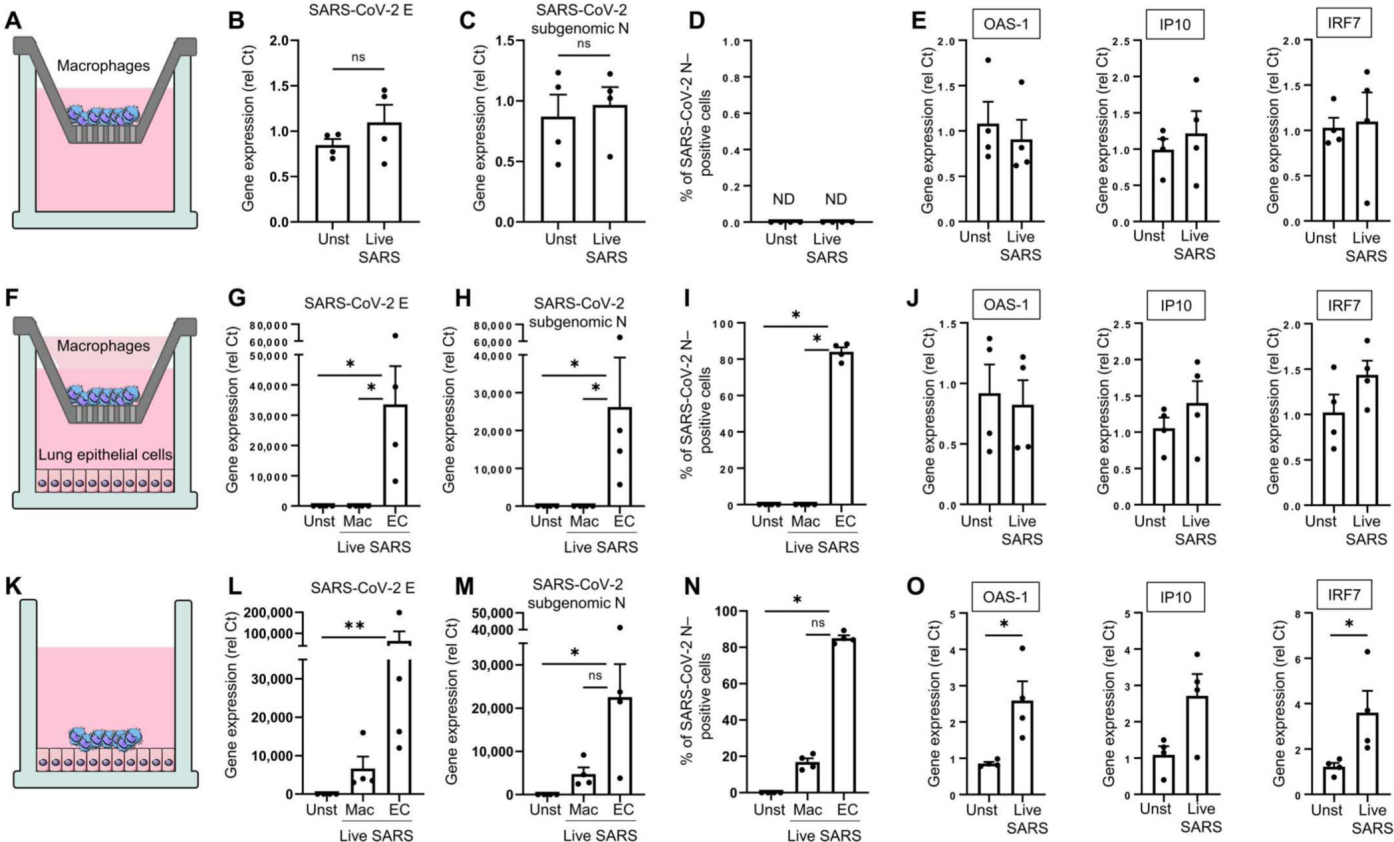
Lung macrophages are indirectly infected by SARS-CoV-2 via the phagocytosis of infected epithelial cells. (**A** to **E**) Alveolar macrophages isolated from human primary lung tissue (Alveo-Macs) were infected or not [unstimulated (Unst)] by live SARS-CoV-2 (MOI = 0.01) for 24 hours in the upper chamber of a transwell. Gene expression of SARS-CoV-2 E (B) and N (C) and ISGs (D) was quantified by qPCR. (**F** to **J**) Alveo-Macs and live SARS-CoV-2–infected lung epithelial cells (MOI = 0.01) were cultured in the upper and lower chambers of the transwell, respectively. After 24 hours, CD68 beads were used to isolate macrophages (Mac; CD68^+^) and epithelial cells (EC; CD68^−^), and gene expression of SARS-CoV-2 E (G) and subgenomic N (H) and the percentage of SARS-CoV-2 N (I)–positive cells were quantified. (G to L) Expression of ISGs was quantified in macrophages (CD68^+^ cells) by qPCR (J). (**K** to **O**) Alveo-Macs and live SARS-CoV-2–infected lung epithelial cells (MOI = 0.01) were cultured together for 24 hours. After the use of CD68 beads, gene expression of SARS-CoV-2 E (L) and subgenomic N (N) and the percentage of SARS-CoV-2 N–positive cells were detected in both macrophages (CD68^+^) and epithelial cells (EC; CD68^−^). Expression of ISGs was quantified in macrophages (CD68^+^ cells) by qPCR (O). All results are represented as means ± SEM. Statistical significance was evaluated using a Friedman test with Dunn’s multiple comparisons posttest or a Mann-Whitney test. *P < 0.05 and **P < 0.01. ND, not determined.

We recently described that less than 10% of lung macrophages are infected with SARS-CoV-2 (8). However, macrophages produce some IFN-I due to the activation of the cGAS-STING pathway (31), because the deletion of STING in a mouse model of SARS-CoV-2 infection partially reduced IFN-I and ISGs (interferon-stimulated genes) expression. These authors did not observe that macrophages can be directly stimulated by SARS-CoV-2. Here, we observed that neither CD14^+^ monocytes, pluripotent stem cell (PSC)–derived macrophages, monocyte-derived macrophages, nor primary alveolar macrophages isolated from human lungs could directly be infected or stimulated by live SARS-CoV-2 (Fig. 5, A to E, and fig. S9, A and B), and inactivated SARS-CoV-2 induced little to no tumor necrosis factor (TNF) or IL-6 in macrophages (fig. S9, C and D). We observed a similar lack of response when alveolar macrophages were cultured with epithelial cells in the presence of live SARS-CoV-2 in a transwell system (Fig. 5, F to J). However, when cultured in combination with epithelial cells (Fig. 5K), genomic SARS-CoV-2 E (Fig. 5L) and SARS-CoV-2 subgenomic N (Fig. 5, M and N) were detected in the macrophages, which then expressed ISGs (Fig. 5O). This suggests that the source of viral RNA and proteins in macrophages is likely not direct infection but phagocytosis of infected cells. Combined with our in vitro data of live SARS-CoV-2–inoculated pDCs, these data support a scenario where IFN-I was coming from at least two different sources: pDCs by direct sensing of the live virus and macrophages by interacting with epithelial cells infected by the virus.

### The production of IFN-I by pDCs in response to SARS-CoV-2 exacerbates macrophage responses to environmental stimuli

**Fig. 6.**
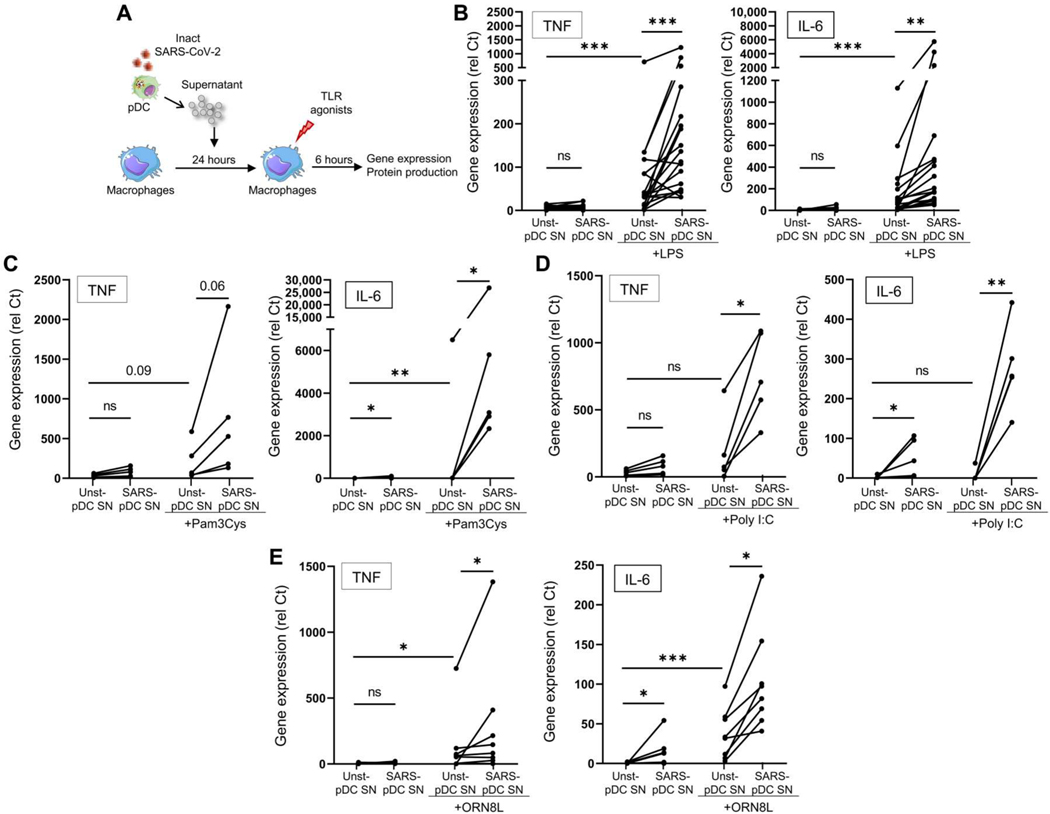
SARS-CoV-2–activated pDCs exacerbate TLR signaling in macrophages. (**A**) Macrophages purified from PBMCs of HDs were cultured for 24 hours with the supernatant from either unstimulated pDCs (Unst-pDC SN) or inactivated SARS-CoV-2–stimulated pDCs (SARS-pDC SN), followed by the addition of TLR agonists for 6 hours. (**B** to **E**) Macrophages purified from PBMCs of HDs (*n* = 5 to 20) were preincubated for 24 hours with the supernatant of Unst-pDC SN or SARS-pDC SN alone or followed by the addition of (B) LPS (10 ng/ml), (C) Pam3Cys (20 ng/ml), (D) poly I:C (10 μg/ml), or (E) ORN8L (60 μM) for 6 hours. Expression levels of TNF and IL-6 were quantified by qPCR. All results are represented as means ± SEM. Statistical significance was evaluated using a Friedman test with Dunn’s multiple comparisons posttest or a Mann-Whitney test. *P < 0.05, **P < 0.01, and ***P < 0.001.

An increase in bacterial infections in patients with COVID-19, resulting in higher levels of bacterial products [bacterial DNA/RNA, lipoproteins, and lipopolysaccharide (LPS)] in intensive care unit (ICU) patients, has recently been reported (56). We therefore investigated whether the activation of macrophages by bacterial or environmental products could be influenced by IFN-I produced by pDCs activated by SARS-CoV-2. Hence, we incubated macrophages overnight with the supernatant of SARS-CoV-2–activated pDCs, and the cells were then cultured in the presence of various pathogen products (Fig. 6A). First, the supernatants of SARS-CoV-2–activated pDCs had little effect when used alone (Fig. 6, B to E). Similarly, supernatants from SARS-CoV-2–activated pDCs had little effect on the ability of the macrophages to respond to SARS-CoV-2 itself (fig. S9, E and F). However, these supernatants drastically amplified the production and expression of proinflammatory cytokines, such as TNF and IL-6, by macrophages in response to not only LPS (Fig. 6B and fig. S10A) but also Pam3Cys (an agonist of another transmembrane TLR) (Fig. 6C and fig. S10B), poly I:C (polyinosinic:polycytidylic acid) (Fig. 6D and fig. S10C), and the TLR8 agonist ORN8L (both RNA-sensing TLRs, which are endosomal) (Fig. 6E and fig. S10D). As control, we used the supernatant of pDCs that were left unstimulated, which had little to no effect on macrophage responses to these stimuli (Fig. 6, B to E). *CXCL10* was induced in macrophages by SARS-CoV-2–infected pDCs, likely due to the presence of IFN-I in the pDC supernatants, and the addition of LPS had little effect (fig. S10E). However, live virus in the context of the inflammatory milieu of the lung may enhance the sensing and inflammatory response by macrophages. In addition, although lung macrophages are not abundantly infected by SARS-CoV-2 (8), we showed that macrophages could be activated by the phagocytosis of infected cells (Fig. 5O) and could also be activated via cell-to-cell transfer of viruses by pDCs as reported in the context of HIV (57). pDCs could respond differently to free particles versus infected cells as previously suggested (40, 58).

**Fig. 7.**
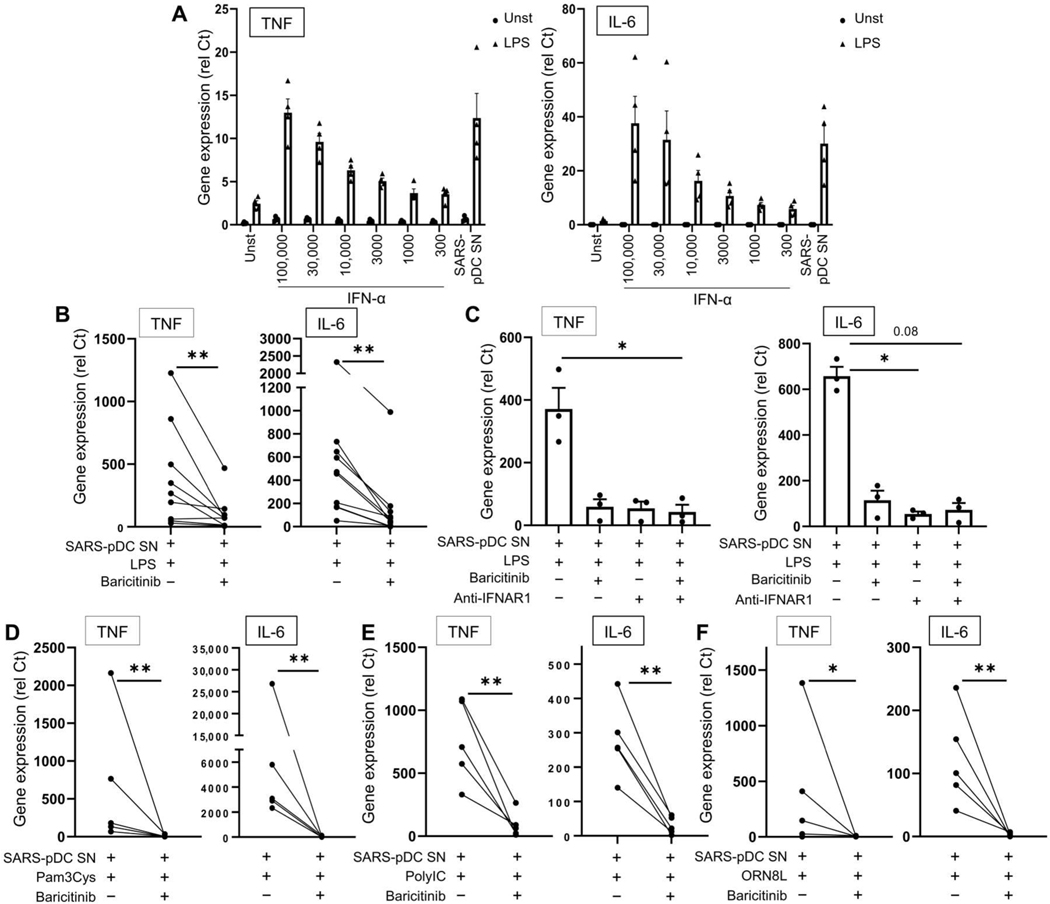
SARS-CoV-2–activated pDCs exacerbate TLR signaling in macrophages via the IFN-I pathway. (**A**) Macrophages purified from PBMCs of HDs (*n* = 4) were preincubated alone (Unst) or with either different concentrations of IFN-α as indicated (picograms per milliliter) or the supernatant of inactivated SARS-CoV-2–stimulated pDC (SARS-pDC SN), followed by the addition of LPS for 6 hours. Expression levels of TNF and IL-6 were quantified by qPCR. (**B** and **C**) Macrophages purified from PBMCs of HDs (*n* = 3 to 10) were preincubated for 24 hours with the supernatant of SARS-pDCs in the presence of baricitinib (2 μM) and/or anti-IFNAR antibody (2 μg/ml), followed by the addition of LPS for 6 hours. Expression levels of TNF and IL-6 were quantified by qPCR. (**D** to **F**) Macrophages purified from PBMCs of HDs (*n* = 5) were preincubated for 24 hours with the supernatant of SARS-pDCs in the presence of baricitinib (2 μM), followed by the addition of (C) Pam3Cys, (D) poly I:C, or (E) ORN8L for 6 hours. Expression levels of TNF and IL-6 quantified by qPCR. (F) All results are represented as means ± SEM. Statistical significance was evaluated using a Friedman test with Dunn’s multiple comparisons posttest or a Mann-Whitney test. *P < 0.05 and **P < 0.01.

Because pDCs produced large amounts of IFN-I in response to SARS-CoV-2 (Fig. 3A), we incubated macrophages with titrated amounts of IFN-α followed by LPS. We observed a dose-dependent effect of IFN-α on the LPS-induced response in macrophages with induction of *TNF*, *IL6*, *IL1B*, *IL12B*, and *IFNB* after treatment with high concentrations of IFN-α, comparable to that observed with supernatants from SARS-CoV-2–infected pDCs (Fig. 7A and fig. S11). We also explored the effect of TNF, which could have a synergistic or antagonistic effect on IFN-α. However, blocking TNF or the TNF receptor had no significant influence on macrophages primed with supernatants from SARS-CoV-2–infected pDCs in response to LPS (fig. S12), although it is possible that TNF may play a role in the context of inflamed lung, where TNF is abundantly secreted by the macrophages. Baricitinib inhibits Janus kinase 1/2 (JAK1/2), which is essential, although not restricted, to IFNAR signaling (59), and early evaluation in combination with remdesivir in hospitalized patients with COVID-19 yielded promising results, with significantly reduced mortality (60). In the absence of LPS, baricitinib prevented the induction of not only *CXCL10* but also TNF and IL-6 secretion in macrophages cultured with the supernatant of SARS-CoV-2–infected pDCs (fig. S13, A and B). Inhibiting JAK1/2 prevented the induction of TNF and IL-6 by LPS (Fig. 7B and fig. S14A). Blocking IFNAR had a similar effect (Fig. 7C and fig. S14B), suggesting that the main effect was due to IFN-I, although it is also possible that other factors secreted by pDCs, beyond IFN-I or IFN-III, may contribute at some level to the modulation of macrophage response. A similar observation was made using Pam3Cys (Fig. 7D and fig. S14C), poly I:C (Fig. 7E and fig. S14D), or ORN8L (Fig. 7F and fig. S14E) in macrophages exposed to SARS-CoV-2–infected pDC supernatants (Fig. 7, B to F, and fig. S14, A to D). We conclude that macrophages produced exacerbated amounts of proinflammatory cytokines in response to environmental stimuli when exposed to IFN-I from SARS-CoV-2–infected pDCs.

### The production of IFN-I by pDCs in response to SARS-CoV-2 mediates epigenetic and transcriptional changes in macrophages

**Fig. 8.**
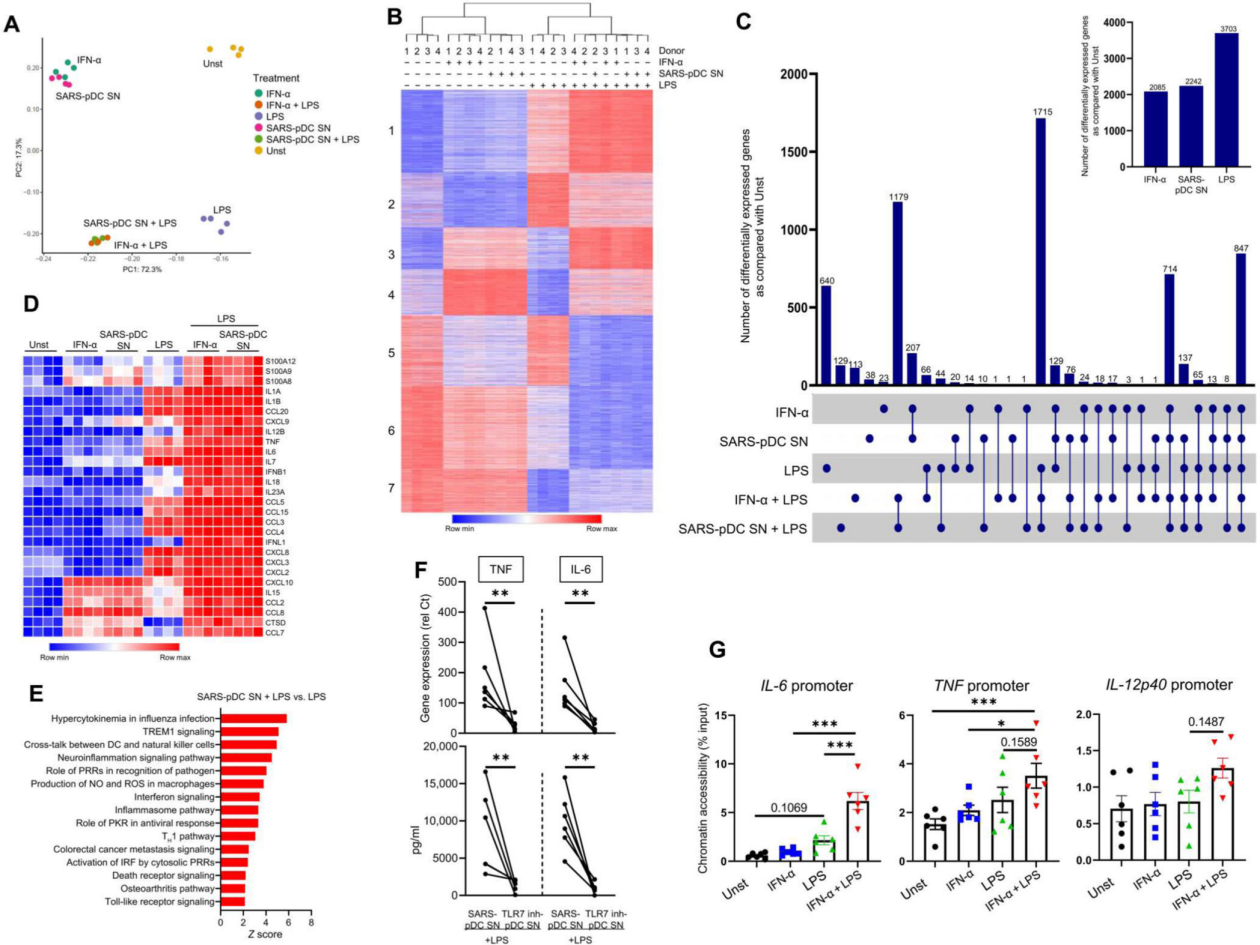
IFN-α increases inflammatory transcription and chromatin accessibility in macrophages. (**A**) PCA of the differentiated genes in either unstimulated (Unst), IFN-α–stimulated macrophages, or SARS-pDC SN–stimulated macrophages, followed by the addition of LPS for 3 hours (2 ng/ml) when indicated. PC1 and PC2 capture percent variation associated with either individual or combination treatments. (**B**) *K*-means clustering (*K* = 7) of DEGs induced by a greater than twofold change with FDR < 0.05 under the conditions shown in (A). (**C**) Macrophages purified from PBMCs of HDs (*n* = 4) were incubated for 24 hours with either Unst-pDC SN, IFN-α, or SARS-pDC SN either alone or followed by the addition of LPS. The number of genes differentiated by IFN-α, SARS-pDC SN, or LPS was normalized to that of the unstimulated condition. (**D**) Heatmap showing the inflammatory genes related to COVID-19 in macrophages incubated under the same conditions as in (A). (**E**) Top activated pathways of the differentiated genes induced by more than twofold, with FDR < 0.05 in macrophages preincubated with SARS-pDC SN and then cultured for 3 hours with LPS versus LPS alone. (**F**) Macrophages purified from PBMCs of HDs (*n* = 6) were cultured for 24 hours with either SARS-pDC SN alone or SARS-pDC SN with the TLR7 inhibitor IRS661 followed by the addition of LPS for 6 hours. Gene expression levels and production of TNF and IL-6 were quantified by qPCR and ELISA, respectively. (**G**) Macrophages purified from PBMCs of HDs (*n* = 6) were incubated alone (Unst) or with IFN-α for 24 hours. LPS was then added (when indicated) for 3 hours, and the FAIRE assay was performed on the promoter regions of IL6, TNF, and IL12p40. Results are represented as means ± SEM. Statistical significance was evaluated using a Mann-Whitney test and one-way ANOVA. *P < 0.05, **P < 0.01, and ***P < 0.001.

To obtain a comprehensive understanding of how IFN-I and SARS-CoV-2–infected pDC supernatants influenced macrophage activation, we performed transcriptomic analysis using RNA-seq to evaluate TLR4 responses in macrophages exposed to IFN-α or SARS-CoV-2–infected pDC supernatants. Principal components analysis (PCA) showed that pDC supernatants and IFN-α conditions closely clustered together (Fig. 8A); IFN-α– and SARS-CoV-2–infected pDC supernatant–induced genes [differentially expressed genes (DEGs), false discovery rate (FDR) < 0.05 and fold induction > 2] highly overlapped with commonly induced genes (Fig. 8, B and C). Similarly, macrophages preincubated with either IFN-α or SARS-CoV-2–infected pDC supernatants and stimulated with LPS tightly clustered together, separated from the LPS-alone condition (Fig. 8A); 92% of DEGs induced by LPS in IFN-α– or SARS-CoV-2–infected pDC supernatant–treated macrophages were common (Fig. 8C). The data showed that both LPS and IFN-α/SARS-CoV-2–infected pDC supernatants contributed to the changes in gene expression (Fig. 8C). Several chemokines are present in the lungs of SARS-CoV-2–infected patients (61). We observed that IFN-α or supernatants from SARS-CoV-2–activated pDCs up-regulated a series of chemokine receptors, including CCR2, CCR1, CCR5, and CXCR2 (fig. S15A). These recognize CCL2, CCL5, CCL8, and CXCL8, which are induced by SARS-CoV-2 or IFN-α (fig. S7, B and C), suggesting a role for IFN-α in promoting the infiltration of macrophages to the lungs of patients. We conducted a *K*-means clustering based on genes with more than a twofold change in expression, which segregated DEGs into seven groups based on patterns of expression (Fig. 8B). The resulting clusters faithfully followed the culture conditions, except for the IFN-α + LPS and SARS-CoV-2–infected pDC supernatant + LPS, which, as expected, yielded similar gene patterns (see top part of Fig. 8B). We conducted pathway analysis of each cluster that segregated the clusters with a strong inflammatory bias (see fig. S15B). Clusters 3 and 4 were dominated by genes induced by IFN-I, whereas clusters 6 and 7 were dominated by genes down-regulated by LPS. Cluster 1 was composed of LPS-inducible genes whose expression was exacerbated by either IFN-α or SARS-CoV-2–infected pDC supernatants (similar to *TNF* and *IL6*). A more in-depth analysis indicated that cluster 1 included many genes encoding proinflammatory cytokines and chemokines (Fig. 8D and fig. S15B) and fibrosis-related genes (fig. S15C), which are implicated in COVID-19 pathogenesis (6, 26, 27). Additional bioinformatic analysis comparing macrophages stimulated with SARS-CoV-2–infected pDC supernatants versus LPS alone revealed enrichment for inflammatory and immune pathways (Fig. 8E and fig. S15D). Ingenuity Pathway Analysis suggested a role for IFN regulatory factor (IRF) and nuclear factor κB (NF-κB) family transcription factors in the activation of inflammatory genes by IFN-α or SARS-CoV-2–infected pDC supernatants with LPS (fig. S15E), which is in line with our previous report (62). Clusters 3 and 4 showed enrichment of IFN signaling (fig. S15B) and were composed of canonical ISGs (fig. S15F). Overall, these results support a role for pDC-derived IFN-I in the exacerbation of TLR4-mediated inflammatory response by macrophages. Accordingly, inhibition of IFN-I production using either the TLR7 inhibitor IRS661 or the PI3Kδ inhibitor CAL-101 reduced inflammatory gene induction upon challenge with LPS (Fig. 8F and fig. S16A). IFN-α had minimal effects on TLR4-induced IκBα degradation or activation of mitogen-activated protein kinases (MAPKs) ERK (extracellular signal–regulated kinase) and p38 (fig. S16B), which is in accord with previous work (62). However, cells primed with IFN-α followed by LPS treatment significantly increased chromatin accessibility of *IL6* and *TNF* promoters (Fig. 8G). Hence, these data demonstrate that the IFN-I produced by pDCs in response to SARS-CoV-2 mediates transcriptional and epigenetic changes in macrophages, which exacerbates their production of inflammatory mediators in response to environmental triggers.

## DISCUSSION

The immune response to SARS-CoV-2 evolves over time in patients with COVID-19 (3). Here, we showed an unexpected role of pDCs during the course of the disease. pDCs were the main producers of IFN-I in the blood and directly sensed SARS-CoV-2 via TLR7. We also observed that macrophages could produce IFN-I due to the phagocytosis of SARS-CoV-2–infected epithelial cells. The entry of SARS-CoV-2 in pDCs required clathrin-mediated endocytosis, and the IFN-I produced by pDCs markedly exacerbated the response of macrophages to various innate stimuli. In the lungs of patients, a peak of pDC infiltration was associated with an initial wave of IFN-I in the macrophages of patients that did not produce a cytokine storm. In contrast, in patients with severe or even fatal COVID-19, IFN-I and proinflammatory signals were both present in the lung-infiltrating macrophages. Our data thus support a model (see fig. S17) by which the IFN-I produced by pDCs due to the direct sensing of SARS-CoV-2 could epigenetically prime lung macrophages to induce a cytokine storm. This model is consistent with the recent findings that macrophages can induce IFN-α via the cGAS-STING pathway but only in response to SARS-CoV-2–infected cells (31). Another model is that an initial defect in pDC response can favor the infection of epithelial cells, which, in turn, can activate the macrophages. This is supported by data in IFN- or TLR7-defective patients (21, 41) and by some studies linking the IFN-I produced by macrophages to disease (31, 63). The macrophage priming could also be done by the presence of IFN-γ, which, by complementing by TLR signaling, promotes caspase-8 pathways leading to cell death and increased severity of SARS-CoV-2 disease (64). Together, this would indicate a redundancy in the response to SARS-CoV-2 by different cell types stimulated via different nucleic acid sensors and the contribution by both IFN-I and IFN-II.

It is difficult to assess the precise amount of IFN-I in the lungs of patients, but pDCs are the highest blood producers at the per-cell level of IFN-α in response to viruses (34, 35, 65), which may create a local environment where very high concentrations of IFN-I are present, even with a limited number of cells (66). Low concentrations of IFN-I (200 antiviral units/ml) induced antiviral ISG expression (“IFN signature” and, by inference, an antiviral response) and did not augment subsequent TLR responses, whereas higher concentrations of IFN-α prevented TNF-induced tolerance of a subset of TLR4-inducible genes (62). Here, extraordinarily high concentrations of IFN-I produced by SARS-CoV-2–infected pDCs reprogrammed macrophages for an augmented hyperinflammatory TLR response. In other words, low concentrations of IFN-I induce antiviral responses without inflammatory toxicity, but high amounts of IFN-I produced by pDCs additionally promote cytokine storm. Because the precise pathogenesis of COVID-19 is still unresolved, it is likely to vary between patients with respect to the source of IFN-I in the lungs. However, our data indicate that pDCs sense SARS-CoV-2 through TLR7, and they were the dominant producer of IFN-I in the blood, a property not shared with other viruses, such as Flu. Using a controlled setting in vitro, we demonstrated that macrophages exposed to SARS-CoV-2–infected pDCs had an exacerbated response to multiple stimuli, directly linking the IFN-I produced by SARS-CoV-2–infected pDCs and the observed hyperactivation of macrophages in patients with COVID-19. Because we showed that the IFN-I response was concentrated in monocyte-derived macrophages in the lungs of patients, our data suggest that, in addition to being primed in the lungs, some macrophages may be primed in the blood before the cells enter the lungs of infected individuals. After IFN priming, exacerbation of activation was observed after stimulation by different TLR ligands. Increased bacterial infections in ICU patients (56) and increased presence of intestinal mucosal damage in patients with COVID-19 (67, 68) may be responsible for the presence of LPS and other TLR ligands and thus induce hyperactivation. It seems that there is no clear correlation between viral loads (often quantified in the blood) and the inflammatory response in the lung [see the review (69)].

Our study has some limitations in part due to the difficulties in working with samples from infected patients. The lung samples were taken from recently deceased individuals with severe diseases, which may explain the low abundance of pDCs (70), consistent with the BAL data (4). These patients are at different time points in their COVID-19 illness, and samples can only be obtained at the time of death. A proper longitudinal time course would be ideal to address the question of pDC numbers over time but could not be done here. Furthermore, we used chemical inhibitors to study the role of TLR7 and the clathrin-mediated pathways, which brings some limitations to our system, including potential off-target effects and redundancy in the pathways.

Our findings have potential translational significance, and the physiological relevance of the pDC-macrophage circuit is supported by the efficacy of the JAK inhibitor baricitinib in decreasing mortality in patients with COVID-19 (60), likely by attenuating the cytokine storm. Our data describe a strong fibrotic signature particularly in BAL macrophages of patients with mild disease, which correlated with IFN-I response. This is consistent with the observation that macrophages have a profibrotic phenotype in patients with COVID-19 (71). Because pDCs can promote fibrosis in systemic sclerosis, it is possible that the pDC-macrophage interaction contributes to this phenotype in COVID-19 as well. In IFN-primed macrophages, JAK inhibitors suppress the expression of canonical inflammatory target genes such as TNF and IL-6, whereas in control macrophages, JAK inhibitors only suppress ISG expression (66). Thus, we propose that one mechanism of action of baricitinib in COVID-19 is direct suppression of the inflammatory cytokine genes that drive the cytokine storm. It remains possible that additional factors produced by pDCs, or direct pDC-macrophage interactions, contribute to the complex macrophage phenotype in COVID-19. Furthermore, SARS-CoV-2 activation of inflammasomes is associated with COVID-19 severity, and viral components produce a variegated response with early effects on inflammasome priming and later changes resulting in inflammasome activation and the production of inflammatory factors (72, 73). In addition, the infection by SARS-CoV-2 triggers the inflammasome in macrophages, leading to IL-1β and IL-18 release in the lungs and contributing to pulmonary inflammation (30). These findings suggest that inhibition of the pDC-macrophage circuit could be beneficial for patients with COVID-19.

When pDCs are chronically activated, they produce sustained levels of IFN-I that lead to negative consequences, such as CD4 T cell depletion in HIV-infected patients or the promotion of autoimmunity by promoting skin lesions (34, 35, 74, 75). Our data now suggest that a similar concept may apply during COVID-19, where the chronic activation of pDCs and the IFN-I that they produce can prime uninfected macrophages to produce a cytokine storm in the lungs of patients with COVID-19. Although we are showing that pDCs could be activated by direct sensing of SARS-CoV-2, in the lungs, the cells could also be activated by virus-infected cells (58). The sensing of infected cells by pDCs could lead to higher levels of IFN, which could compensate for the low number of pDCs present in the lungs of patients with severe disease. In addition, blood pDCs from patients with severe COVID-19 have a reduced response to SARS-CoV-2 (76), which is reminiscent of HIV, where the number of pDCs is reduced (77), and of autoimmune indications such as lupus or systemic sclerosis (34) and is an indication that the activated cells may have migrated to the tissue.

Although the cytokines storm produced in the lungs by macrophages is responsible for respiratory distress and poor outcome in SARS-CoV-2–infected patients, the underlying mechanism leading to the activation of macrophages is not well defined. Furthermore, whether IFN-I contributes to the inflammatory response in the lung is also unclear. Our data describe that pDCs and the IFN-I that they produce in response to SARS-CoV-2 can exacerbate the response of macrophages to innate stimuli that these cells can encounter in the lungs. This identifies the pDC-macrophage circuit as critical for the pathogenesis of COVID-19 and identifies the TLR7 sensing of SARS-CoV-2 by pDCs as a central element of the immune response to this virus. Hence, pDCs and TLR7 are needed to provide an adequate response to control the virus, but the IFN-I induced in pDCs can also exacerbate macrophage response, with deleterious consequences. These data also have translational potential because they identify pDCs and the IFN-I pathway as potential therapeutic targets for critically ill patients with COVID-19.

## MATERIALS AND METHODS

### Study design

The research objective of our study was to determine how pDCs, through IFN-I production, might contribute to the pathogenesis of COVID-19 by activating lung macrophages. Patients with COVID-19 and healthy donors were included in the human study. Patients have been described in earlier studies (4, 6). Control samples were from the New York Blood Center (Long Island City, NY). We screened both BAL (GSE145926) and lung (GSE171524) to identify pDC-associated IFN-I responses and COVID-19 inflammatory genes in lung macrophages of patients. We studied the priming of macrophages by SARS-CoV-2–activated pDCs by incubating macrophages with the supernatant of SARS-CoV-2–activated pDCs followed by the addition of lung environmental products. The mechanism of this priming was determined using anti-IFNAR antibody. We identified that lung macrophages could also be activated by phagocytosis of infected epithelial cells by doing a coculture experiment. RNA-seq analysis and formaldehyde-assisted isolation of regulatory element (FAIRE) assays in macrophages incubated with SARS-CoV-2–activated pDC supernatant and then LPS revealed changes at transcriptional and epigenetic levels. No blinding or randomization was performed for the human studies. Raw data are available in table S6.

### Purification of cells and cell lines

Enriched leukocytes were obtained from the New York Blood Center (Long Island City, NY) after informed consent of donors who were deemed healthy by the New York Blood Center’s criteria and used under a protocol approved by the Institutional Review Board of the Hospital for Special Surgery and the Institutional Biosafety Committee of Weill Cornell Medicine (2014–221). PBMCs were prepared using Ficoll-Paque density gradient (GE Healthcare) as previously described (78). pDCs and monocytes were isolated from PBMCs by positive selection using BDCA4-conjugated (78) and CD14-conjugated (79) microbeads (Miltenyi Biotec), respectively. pDC-depleted PBMCs were prepared by removing BDCA4-positve cells from PBMCs using microbeads (Miltenyi Biotec). Macrophages were differentiated from monocytes by culturing in complete RPMI 1640 medium with macrophage colony-stimulating factor (M-CSF; 20 ng/ml) for 1 to 5 days. Vero E6 cells [African green monkey (*Chlorocebus aethiops*) kidney] were obtained from the American Type Culture Collection (ATCC). A549 cells (human adenocarcinomic alveolar basal epithelial) were obtained from ATCC. A549-ACE2 cells were obtained from B.R.t. (Mount Sinai).

### Primary human pulmonary cells

Human alveolar epithelial cells were obtained from ScienCell, cultured in alveolar epithelial cell medium (ScienceCell), expanded in T-25 plates, and plated in Transwell plates (Corning) for additional experiments. Human alveolar macrophages were obtained from AcceGen and cultured in complete RPMI 1640 medium with M-CSF (100 U/ml), 1 mM sodium pyruvate, 10 mM Hepes, and 1× penicillin/streptomycin.

### Human PSC culture

The human induced PSCs (iPSCs) were grown and maintained on 1% Matrigel-coated six-well plates in mTeSR (STEMCELL Technologies) with 5% CO_2_ culture condition. The medium was changed daily. When iPSCs reached ~90% confluence, the cells were passaged at 1:6 to 1:12 with Accutase (STEMCELL Technologies).

### Human PSC differentiation

Human iPSCs were differentiated into monocytes and macrophages as previously reported (80). In brief, iPSCs were treated with Accutase, scraped to produce small cellular groups, and replated onto 1% Matrigel-coated six-well plates. After 1 to 2 days, mTeSr medium was replaced with mesoderm induction medium containing activin A (15 ng/ml; R&D Systems), bone morphogenetic protein 4 (BMP-4; 40 ng/ml; R&D Systems), and 1.5 μM CHIR99021 (STEMCELL Technologies). On day 2, the medium was replaced with hemogenic endothelium induction containing IL-6 (50 ng/ml; R&D Systems), IL-3 (15 ng/ml; R&D Systems), TPO (thrombopoietin, 50 ng/ml; R&D Systems), basic fibroblast growth factor (bFGF; 12.5 ng/ml; PeproTech), stem cell factor (SCF; 50 ng/ml; R&D Systems), and vascular endothelial growth factor (VEGF; 50 ng/ml; R&D Systems). On day 5, the medium was replaced with hematopoietic induction medium containing VEGF (50 ng/ml; R&D Systems), bFGF (50 ng/ml), SCF (50 ng/ml; R&D Systems), and 10 μM SB431542 (Tocris). On day 9, the cells were dissociated with Accutase and resuspended with monocyte induction medium containing IL-6 (50 ng/ml; R&D Systems), IL-3 (12 ng/ml; R&D Systems), and M-CSF (80 ng/ml; R&D Systems) into low-attachment plates. On day 15, floating cells were collected and the StemSep Human CD14 Positive Selection Kit (STEMCELL Technologies) was used to isolate CD14^+^ cells. Macrophages were obtained by plating monocytes onto fetal bovine serum (FBS)–coated plates with macrophage differentiation medium containing M-CSF. Cells were cultured under normoxic conditions at 37°C and 5% CO_2_.

### Activation of cells

For functional assays, freshly isolated pDCs were resuspended at 3 × 10^4^ cells per 100 ml of complete RPMI and cultured in a 96-well U-bottom plate. PBMCs and pDC-depleted PBMCs were resuspended at 3 × 10^5^ cells per 100 ml of complete RPMI and cultured in a 96-well flat-bottom plate. Untreated and gamma-irradiated inactivated SARS-CoV-2 (USA-WA1/2020, BEI Resources) were used at a multiplicity of infection (MOI) of 1, 0.25, 0.5, 0.1, and 0.01, whereas CpG 274 (TriLink BioTechnologies) was used at 0.5 μM. For blocking experiment, cells were preincubated for 1 hour with the ACE2 inhibitor (2 μM; Novus Biologicals), TLR7 inhibitor IRS661 (2 μM; TriLink BioTechnologies), PI3Kδ inhibitor CAL-101 (10 μM; Selleck Chemicals), clathrin inhibitor chlorpromazine (CPZ; 30 μM; Sigma-Aldrich), or dynamin inhibitor dynasore hydrate (DH; 100 μM; Sigma-Aldrich) followed by the addition of inactivated SARS-CoV-2 at an MOI of 0.25. After differentiation of macrophages, the cells were cultured at 1 × 10^5^ cells per 100 ml of complete RPMI for 24 hours with either 10% of nonactivated pDC supernatant (Unst-pDC SN), 10% of SARS-CoV-2–activated pDC supernatant (SARS-pDC SN), recombinant IFN-α (at 100,000, 30,000, 10,000, 3000, 1000, or 300 pg/ml; PBL Assay Science), or inactivated SARS-CoV-2 (MOI of 0.25). LPS (2 or 10 ng/ml), Pam3Cys (20 ng/ml), poly I:C (10 ng/ml), ORN8L (60 μg/ml), or inactivated SARS-CoV-2 (MOI of 0.25) was added in the culture for 6 hours. For blocking experiments, macrophages were preincubated with the JAK1/2 inhibitor baricitinib (2 μM) or anti-IFNAR antibody (2 μg/ml) for 1 hour before adding SARS-pDC SN for 24 hours. TLR ligands were then added to the well for 6 hours. For TNF blocking, SARS-pDC SN and macrophages were preincubated for 1 hour with anti-hTNF (10 μg/ml; R&D Systems) and antihTNFRI (10 μg/ml; R&D Systems), respectively. After 1 hour, macrophages were incubated with SARS-pDC SN + anti-hTNF for 24 hours. LPS (10 ng/ml) was then added to the well for 6 hours.

### Primary human pulmonary cell coculture

Human alveolar epithelial cells (2 × 10^5^) were plated in T-24 transwell plates (Corning) in Alveolar Epithelial Cell Medium (ScienCell) supplemented with M-CSF (100 U/ml), whereas 5 × 10^4^ human alveolar macrophages were plated in Transwell Plate Inserts in Alveolar Epithelial Cell Medium supplemented with M-CSF (100 U/ml). Cultures were inoculated with mock infection [Dulbecco’s modified Eagle’s medium (DMEM) complemented with 2% FBS, d-glucose (4.5 g/liter), 4 mM l-glutamine, 10 mM nonessential amino acids, 1 mM sodium pyruvate, and 10 mM Hepes] or SARS-CoV-2 (USA-WA1/2020, BEI Resources) at MOI = 0.1. After infections, cells were lysed in TRIzol or fixed in 4% paraformaldehyde for 24 hours. For coculture cells, cells were lifted with trypsin and then fixed in 4% paraformaldehyde for 24 hours. Macrophages were then isolated and separated using antihuman CD68 antibody (STEMCELL Technologies) and StemSep beads (STEMCELL Technologies).

### SARS-CoV-2 propagation and infection

SARS-CoV-2 isolate USA-WA1/2020 (NR-52281) was provided by the Centers for Disease Control and Prevention (CDC) and obtained through BEI Resources, National Institute of Allergy and Infectious Diseases, National Institutes of Health. SARS-CoV-2 was propagated in Vero E6 cells [African green monkey (*C. aethiops*) kidney, ATCC] in DMEM supplemented with 2% FBS, d-glucose (4.5 g/liter), 4 mM l-glutamine, 10 mM nonessential amino acids, 1 mM sodium pyruvate, and 10 mM Hepes using a passage 2 stock of virus as described previously (81). Three days after infection, virus-containing supernatants were purified as described previously (82). In brief, supernatant containing propagated virus was filtered through an Amicon Ultra 15 (100-kDa) centrifugal filter (Millipore Sigma) at ~4000 rpm for 20 min. Flow through was discarded, and the virus was resuspended in DMEM supplemented as described above. Infectious titers of SARS-CoV-2 were determined by plaque assay in Vero E6 cells in minimum essential media supplemented with 2% FBS, 4 mM l-glutamine, 0.2% bovine serum albumin, 10 mM Hepes, 0.12% NaHCO_3_, and 0.7% agar. All MOIs were based on titer determined from plaque assays on Vero E6 cells. All work involving live SARS-CoV-2 was performed in the CDC and U.S. Department of Agriculture–approved BSL-3 (biosafety level 3) facility of the Icahn School of Medicine at Mount Sinai and NYU Langone in accordance with institutional biosafety requirements.

### Influenza infection

PBMCs and pDC-depleted PBMCs were resuspended at 3 × 10^5^ cells/100 ml of complete RPMI and cultured in a 96-well flat-bottom plate. Inactivated influenza virus (H1N1 A/PR/8/34, ATCC) was used at an MOI of 2 for 18 hours. For blocking experiment, cells were preincubated for 1 hour with the TLR7 inhibitor IRS661 (2 μM) followed by the addition of inactivated Flu at an MOI of 2.

### Real-time quantitative PCR analysis

Polymerase chain reactions (PCRs) were performed as described previously with 10 ng of complementary DNA (cDNA) (52). In brief, RNA was extracted from cells using the Qiagen RNeasy Mini Kit. Quantity of RNA was measured by NanoDrop, and the High-Capacity cDNA Reverse Transcription Kit was used to generate 20 to 50 ng of cDNA. Gene expression levels were calculated on the basis of relative threshold cycle (Ct) values. This was done using the formula relative Ct = 100 × 1.8 (HSK-GENE), where HSK is the mean Ct of duplicate housekeeping gene runs (we used Ubiquitin), GENE is the mean Ct of duplicate runs of the gene of interest, and 100 is arbitrarily chosen as a factor to bring all values above 0. Primers were from Fisher Scientific (table S5).

### Real-time quantitative PCR analysis for viral genes

Total RNA samples were prepared from cells using TRIzol and the Direct-zol RNA Miniprep Plus Kit (Zymo Research) according to the manufacturer’s instructions. To quantify viral replication, measured by the accumulation of subgenomic N transcripts, we performed one-step quantitative real-time PCR using the SuperScript III Platinum SYBR Green One-Step qRT-PCR Kit (Invitrogen) with primers specific for the TRS-L and TRS-B sites for the N gene and 18*S* and ACTB as an internal reference as previously described (83). Quantitative real-time PCRs were performed on the Bio-Rad CFX384 Touch Real-Time PCR Detection System. Delta-delta-cycle threshold (DDCT) was determined relative to the 18*S* and ACTB and mock-infected/treated samples.

### Chemokine and cytokine measurement

Supernatant from pDCs or macrophages was used for the quantification of secreted TNF and IL-6 and measured with an enzyme-linked immunosorbent assay (ELISA) according to the manufacturer’s protocol (Mabtech).

### Western blot

Cells were lysed in 50 μl of cold 50 mM tris-HCl (pH 7.4), 150 mM NaCl, 1 mM EDTA, 1% (v/v) Triton X-100, 2 mM Na_3_VO_4_, 1× PhosSTOP EASYPACK, 1 mM Pefabloc, and 1× EDTA-free complete protease inhibitor cocktail (Roche, Basel, Switzerland) and then incubated for 10 min on ice. Then, cell debris was pelleted at 13,000 rpm at 4°C for 10 min. The soluble protein fraction was mixed with 4× Laemmli sample buffer (Bio-Rad, catalog no.1610747) and 2-mercaptoethanol (Sigma-Aldrich). Samples for Western blot were subjected to electrophoresis on 4 to 12% bis-tris gels (Invitrogen). To detect IRF5 dimers, we adopted Novex WedgeWell 14% tris-glycine gel (Invitrogen, catalog no. XP00140BOX) to electrophoresis of protein samples according to the manufacturer’s instruction. Proteins were transferred to polyvinylidene difluoride membrane, and immunodetection was performed as previously published (84). The antibodies used were from Cell Signaling Technology: IκBα (9242s), phospho-p38 (9215S), p38 (9212S), and phospho-p44/42 MAPK (ERK1/2) (9101S).

### FAIRE assay

To evaluate the inflammatory gene in the genome associated with regulatory activity, FAIRE assay was adopted as previously described (84). Briefly, cells were cross-linked with 1% formaldehyde for 15 min and quenched with 0.125 M glycine. Then, cells were lysed and sonicated. Ten percent of the samples was used for input, and the rest was used for phenol/chloroform extraction. The input DNA and extracted DNA were used for qPCR.

### RNA sequencing

After RNA extraction, libraries for sequencing were prepared using the NEBNext Ultra II RNA Library Prep Kit for Illumina following the manufacturer’s instructions (Illumina). Quality of all RNA and library preparations was evaluated with Bioanalyzer 2100 (Agilent), and the sequencing input was 500 ng of total RNA. Sequencing libraries were sequenced by the Genomics Facility at Weill Cornell using NextSeq2000, with 50–base pair paired-end reads at an average depth of 35 million reads per sample.

### RNA-seq analysis

Read quality was assessed, and adapters were trimmed using fastp. Reads were then mapped to the human genome (hg38), and reads in exons were counted against Gencode version 33 (Ensembl 99) with STAR Aligner. Differential gene expression analysis was performed in R (4) using edgeR. Genes with low expression levels (<4 counts per million in at least one group) were filtered from all downstream analyses. The Benjamini-Hochberg FDR procedure was used to correct for multiple testing. Downstream analyses were performed in R using a visualization platform built with Shiny developed by bioinformaticians at the David Z. Rosensweig Genomics Research Center at the Hospital for Special Surgery.

### Analysis of single-nucleus RNA-seq data from the lungs of patients with COVID-19

We used publicly available data of single-nucleus RNA-seq (snRNA-seq) from postmortem lung tissue of patients with COVID-19 (6). We used both the original global UMAP (Uniform Manifold Approximation and Projection) representation and cell type labels provided by authors. Because pDCs were not labeled as a cell type, we proceeded to recluster DCs by recalculating a neighbor graph and computing a UMAP representation with only those cells, using default parameters of Scanpy (version 1.8.1), as described (6). DCs were then clustered using the Leiden algorithm with a resolution of 0.4. The top representative genes for each cluster were obtained using the “rank_genes_groups” function of Scanpy on the original raw expression values and with a *t* test overestimating the variance of each group. Gene expression displayed as an overlay of UMAP plots or in heatmaps represents the raw counts log-transformed after adding the unit value. To test the under- or overrepresentation of cells of either control patients or patients with COVID-19 within each cluster, we used Fisher’s exact test with a two-sided alternative and multiple testing correction using Benjamini-Hochberg’s FDR method in Pingouin (version 0.4.0), as described (6).

To investigate the cellular state of each cell type dependent on COVID-19 infection, we used two sources of gene expression signatures: (i) genes up-regulated in association with inflammation and fibrotic phenotype in COVID-19 found using bulk RNA-seq and (ii) the set of 50 Hallmark pathways from the Molecular Signatures database (version 7.4), as described (6). Each signature was scored in every single cell using the “score_genes” function of Scanpy, and the values were aggregated per cell type and disease state (control/COVID-19) using the mean. The difference between the values in COVID-19 compared with control in each cell type was used as a measure of differential signature activity associated with disease. Furthermore, to estimate the relationship between IFN-α, inflammation, and fibrosis during COVID-19, we calculated the Pearson correlation between these signatures across cells of the same cell type and disease state, for all cell types. This was done with equal cell numbers per cell type in control and COVID-19 groups by randomly sampling the same number of cells. Samples containing fewer than 1000 cells after filtering and samples with low-quality clusters were removed.

### Statistical analysis

GraphPad Prism for Windows was applied for all statistical analysis. The data are shown as means ± SEM. Mann-Whitney test or one-way analysis of variance (ANOVA) was used to assess the significance of difference as indicated in the figure legends, and the following *P* values were applied: *P < 0.05, **P < 0.01, and ***P < 0.001. Detailed information about statistical analysis, including tests and values used, and the number of experiments is provided in the figure legends.

## Supplementary Material

Table S2

Table S1

Table S3

Table S5

Table S4

Table S6

1

## Data Availability

The bulk RNA-seq data are available in the Gene Expression Omnibus database (accession no. GSE182572). BAL data come from DOI:10.1038/s41591-020-0901-9 (GSE145926), and lung biopsy data come from DOI:10.1038/s41586-021-03569-1 (GSE171524).
