# Supplementary material for: Sensing of SARS-CoV-2 by pDCs and their subsequent production of IFN-I contribute to macrophage-induced cytokine storm during COVID-19
Source: Sci Immunol. Author manuscript; Available in PMC 2023 Jan 20. (PMC9853436; doi:10.1126/sciimmunol.add4906)
Supplement: 1 [file NIHMS1843099-supplement-1.pdf]

Science Immunology
AAAS

Supplementary Materials for

Sensing of SARS-CoV-2 by pDCs and their subsequent production of IFN-I contribute to macrophage-induced cytokine storm during COVID-19

Paôline Laurent *et al.*

Corresponding author: Franck J. Barrat, barratf@hss.edu

Sci. Immunol. **7**, eadd4906 (2022)
DOI: 10.1126/sciimmunol.add4906

The PDF file includes:

Figs. S1 to S17
Legends for tables S1 to S6

Other Supplementary Material for this manuscript includes the following:

Tables S1 to S6

Supplementary Materials

Figure S1: scRNA-seq of bronchoalveolar lavage of COVID-19 patients highlights dynamic cell composition and response.

Figure S2: Confirmation of IFN-I as the main driver of disease progression between with an orthogonal approach.

Figure S3: Gene expression and cell cluster in lungs of COVID-19 patients.

Figure S4: Relationship between interferon and inflammation pathways with complement cascades involved in coagulation in lung tissues.

Figure S5: Relationship between interferon and inflammation pathways with complement cascades involved in coagulation in BAL fluid.

Figure S6: pDCs are the dominant producers of IFN-I in response to SARSCoV-2 compared to Flu infection.

Figure S7: SARS-CoV-2 induces chemokines production by infected pDCs.

Figure S8: TLR7 sensing of SARS-CoV-2 by pDCs is necessary for the optimal induction of IFN-α by PBMC, while Flu can use other pathways.

Figure S9: SARS-CoV-2 minimally activate macrophage response.

Figure S10: SARS-CoV-2-infected pDCs exacerbate macrophage activation.

Figure S11: IFN-α induces inflammatory genes in dose-dependent manner.

Figure S12: TNF produced by SARS-CoV-2-infected pDCs doesn't contribute to exacerbation of the macrophage response to LPS.

Figure S13: The activation of macrophage by supernatants of SARS-CoV-2-infected pDCs is blocked by baricitinib.

Figure S14: The induction of macrophage activation by supernatants of SARS-CoV-2-infected pDCs is blocked by baricitinib and requires IFN-I signaling.

Figure S15: IFN-α increases TLR4-induced gene expression but not TLR4-mediated signaling pathway activation.

Figure S16: Supplementary data Figure 14: pDCs require TLR7 signaling and PI3Kδ in response to SARS-CoV-2 in order to superinduce macrophage response to stimuli, while IFN-α does not increase TLR4-mediated signaling pathway activation.

Figure S17: Sensing of SARS-CoV-2 and IFN-I production by pDCs promotes the cytokine storm by macrophages.

Table S1: List of genes associated with IFN-I responses, COVID-19 inflammatory genes and fibrotic genes.

Table S2: IFN-I response, COVID-19 inflammatory genes and fibrotic genes in each cell types from control and COVI-19 patient lung biopsies.

Table S3: List of genes associated with each cluster of DC.

Table S4: Number of cells per donors or COVID-19 patients.

Table S5: List of primers.

Table S6: Raw data.

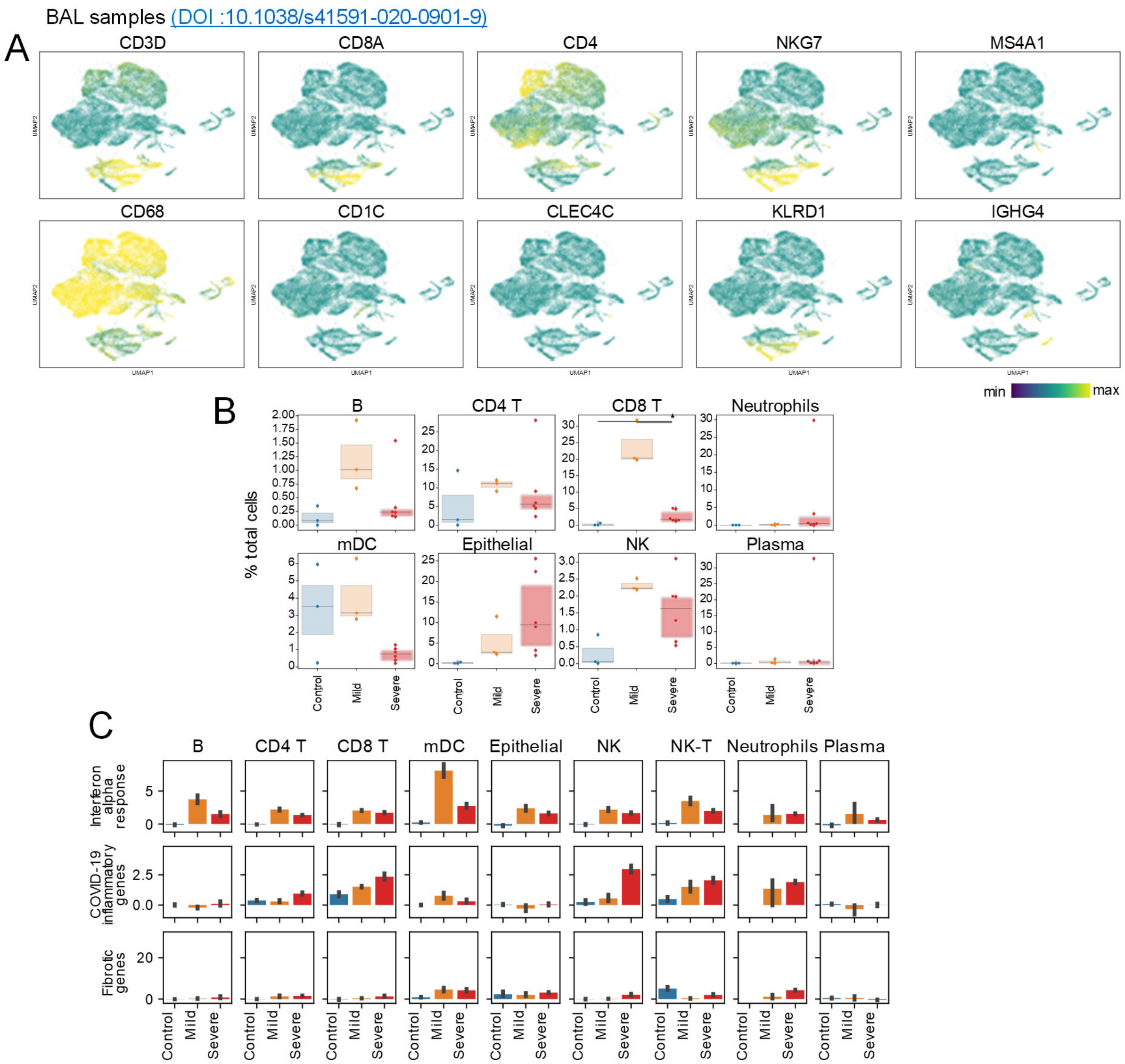

Supplementary figure 1: scRNA-seq of bronchoalveolar lavage of COVID-19 patients highlights dynamic cell composition and response.

(**A**) Expression of cell type defining markers in the UMAP representation of scRNA-seq data from bronchoalveolar lavage (BAL) of COVID-19 patients and non-COVID-19 controls reanalyzed from DOI :10.1038/s41591-020-0901-9. (**B**) Abundance of cell types depending on disease state in BAL. (C) Abundance of gene expression signatures aggregated by cell type and disease state in BAL.

BAL samples (DOI :10.1038/s41591-020-0901-9)

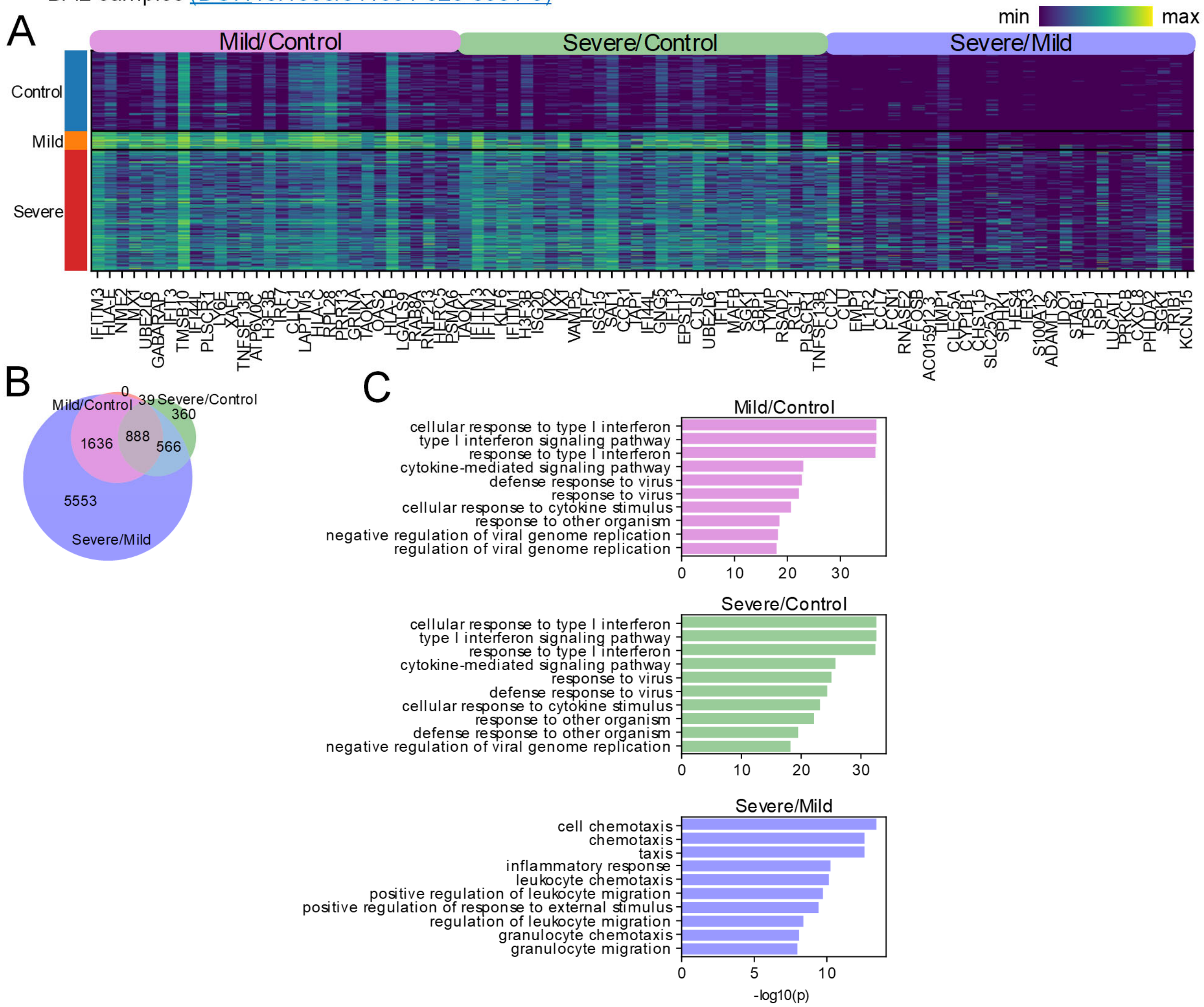

Supplementary figure 2: Confirmation of IFN-I as the main driver of disease progression between with an orthogonal approach.

(**A**) Heatmap of genes differentially expressed in macrophages from BAL, reanalyzed from DOI :10.1038/s41591-020-0901-9, between the two groups of COVID-19 patients and between each group and controls. The top 15 genes were selected per comparison for display. (**B**) Venn diagram of all differentially expressed genes in BAL between disease states. (**C**) Enrichment of the top 100 differentially expressed genes in BAL per disease group comparison in gene ontology terms. We display the top 10 terms for each comparison and FDR-adjusted p-values.

Lung samples (DOI : 10.1038/s41586-021-03569-1)

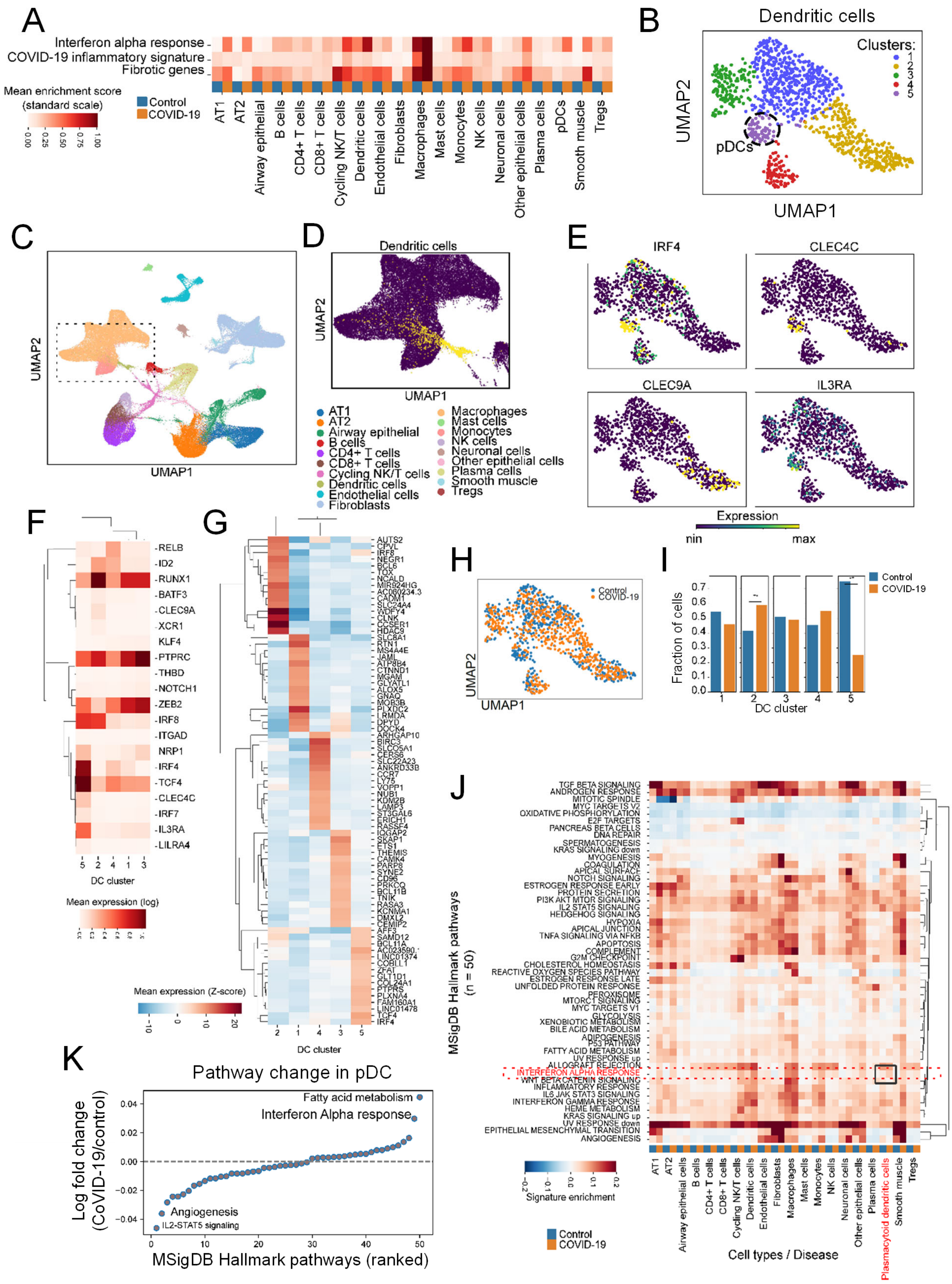

Supplementary figure 3: Gene expression and cell cluster in lungs of COVID-19 patients. (**A**) Heatmap of mean inferred signature activity for all cell types from lung biopsies, reanalyzed from DOI : 10.1038/s41586-021-03569-1, dependent on disease status. Values were min-max scaled per signature to enable comparison. (**B**) UMAP representation of DCs from a scRNA sequencing dataset of lung tissue from COVID-19 patients[4]. Cells are colored by cluster identity, where cluster 5 are pDCs. (**C**) UMAP of scRNA-seq dataset with cells colored by their cell type identity. The area inside the dashed line square is examined in panel B. (**D**) Enlargement of the area highlighted in B (where DCs in yellow). (**E**) Expression of markers specific to clusters of DCs in a new UMAP projection made from DCs only. (**F**) Mean expression of known markers of cell identity or function of DCs across the five clusters of DCs. (**G**) Mean expression of marker genes specific to each DC cluster discovered in an unsupervised manner. The top 10 genes of each cluster are displayed, and their values are Z-score transformed across rows. (**H**) UMAP representation of DCs where cells are colored by the disease state of the donor (Control or COVID-19). (**I**) Proportion of dendritic cells from each cluster that belong to COVID-19 patients or controls in lung tissue. Statistical significance was evaluated with a Fisher's exact test and p-values adjusted for multiple testing with Benjamini-Hochberg false discovery rate method (BH-FDR). (**J**) Heatmap of Hallmark pathway signatures across the major cell types found in lung tissue of Control or COVID-19 patients. (**K**) Pathways ranked by change in inferred activity between COVID-19 and controls in pDCs.

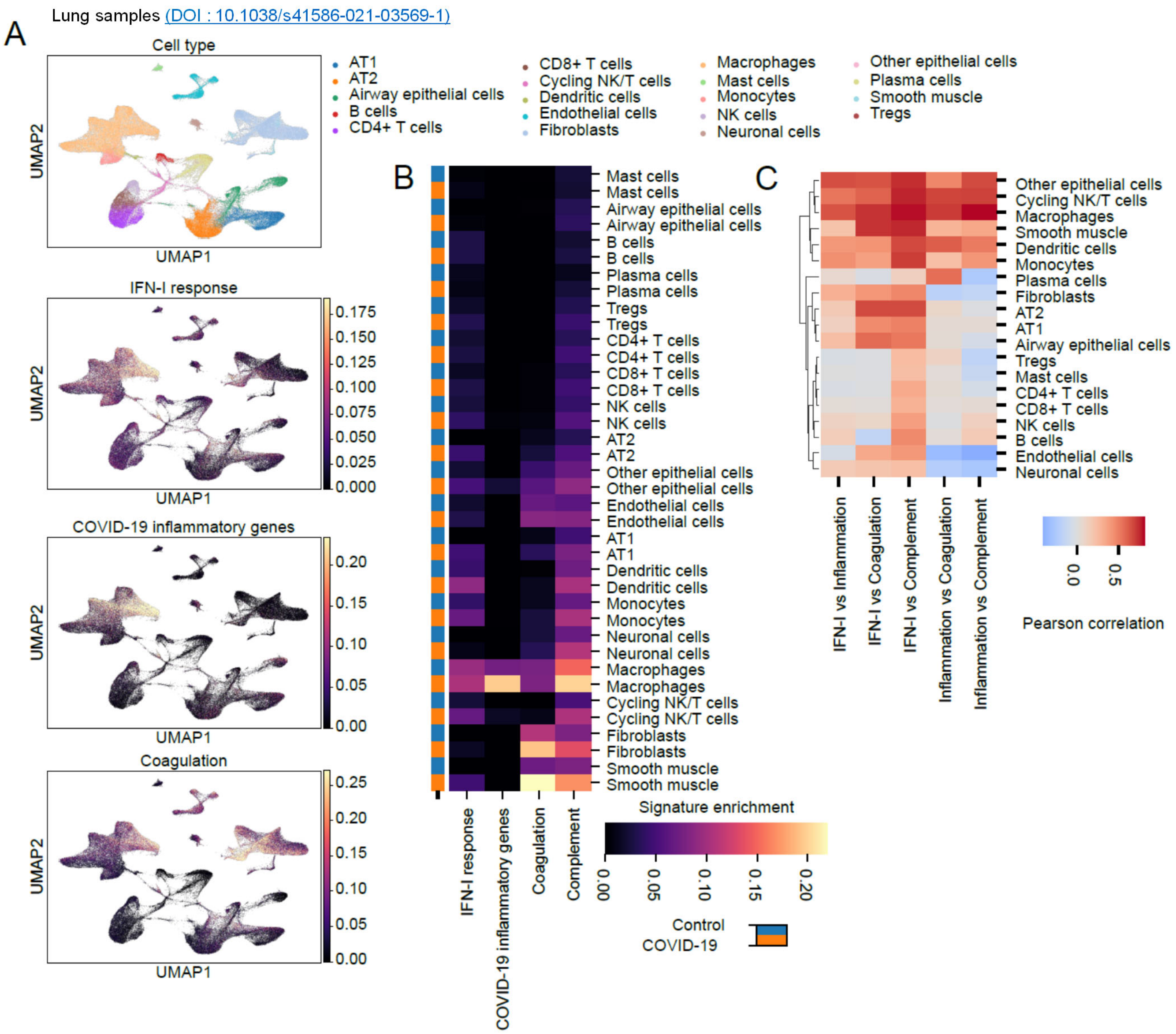

Supplementary figure 4: Relationship between interferon and inflammation pathways with complement cascades involved in coagulation in lung tissues.
(**A**) UMAP representation of a snRNA-seq dataset from lung biopsies of COVID-19 patients, reanalyzed from DOI: 10.1038/s41586-021-03569-1, showing the inferred cell type identities and the intensity of signatures related with IFN-I response, COVID-19 inflammation and coagulation (**B**) Heatmap of mean pathway enrichment score of IFN-I response, COVID-19 inflammation, coagulation and complement per cell type stratified by sample group (control vs COVID-19 patients). (**C**) Heatmap of pairwise correlation between IFN-I response, COVID-19 inflammation, complement and coagulation pathways across single-cells depending on cell type.

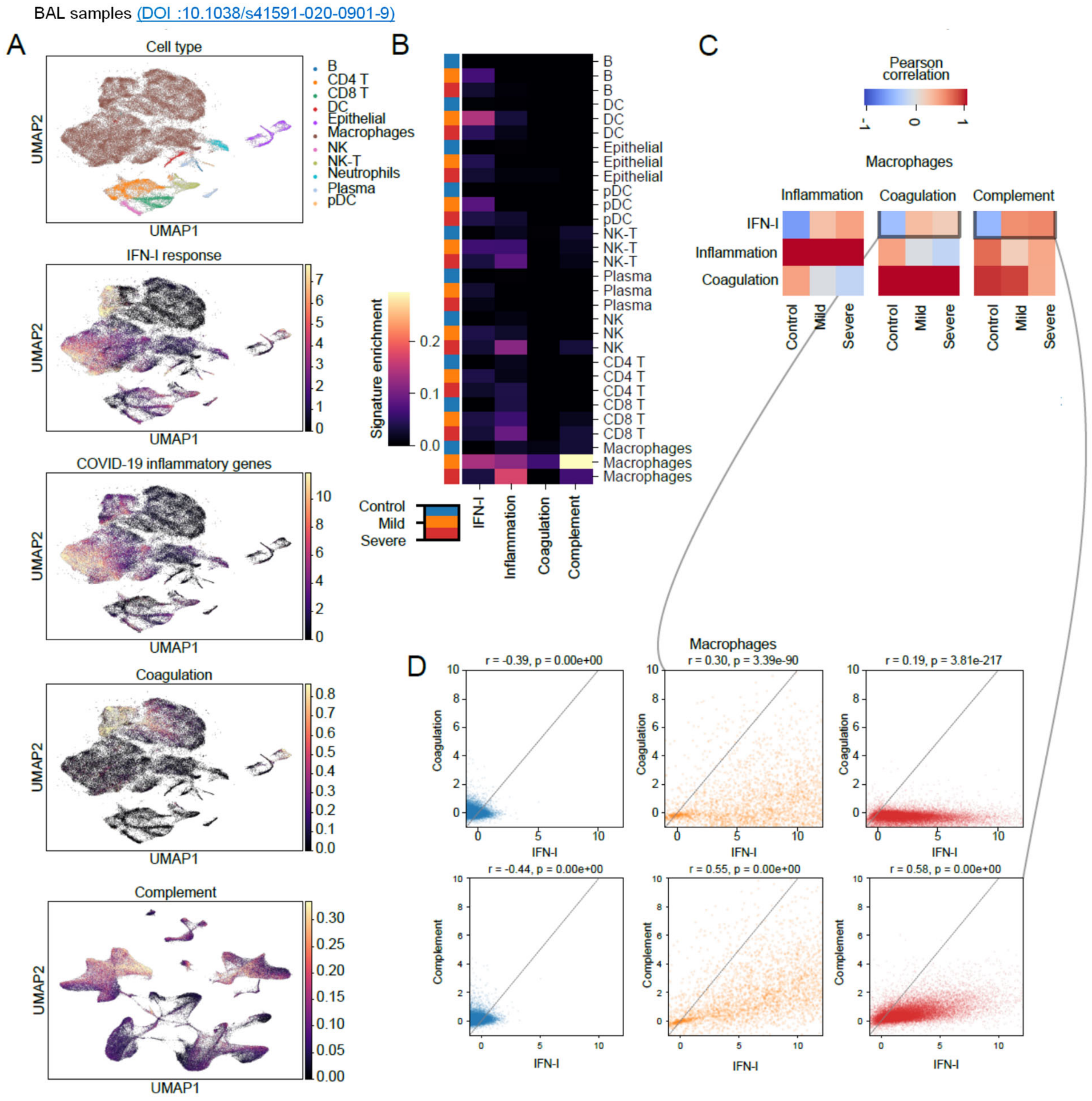

Supplementary figure 5: Relationship between interferon and inflammation pathways with complement cascades involved in coagulation in BAL fluid.

(**A**) UMAP representation of a scRNA-seq dataset from the BAL of COVID-19 patients, reanalyzed from DOI :10.1038/s41591-020-0901-9, showing the inferred cell type identities and the intensity of signatures related with IFN-I response, COVID-19 inflammation, coagulation and complement. (**B**) Heatmap of mean pathway enrichment score of IFN-I response, COVID-19 inflammation, coagulation and complement per cell type stratified by sample group and disease severity. (**C-D**). Heatmap (**C**) or plot (**D**) of pairwise correlation between IFN-I response, COVID-19 inflammation, complement and coagulation pathways across single-cells for macrophages. The Pearson correlation value (r) and p-value are reported.

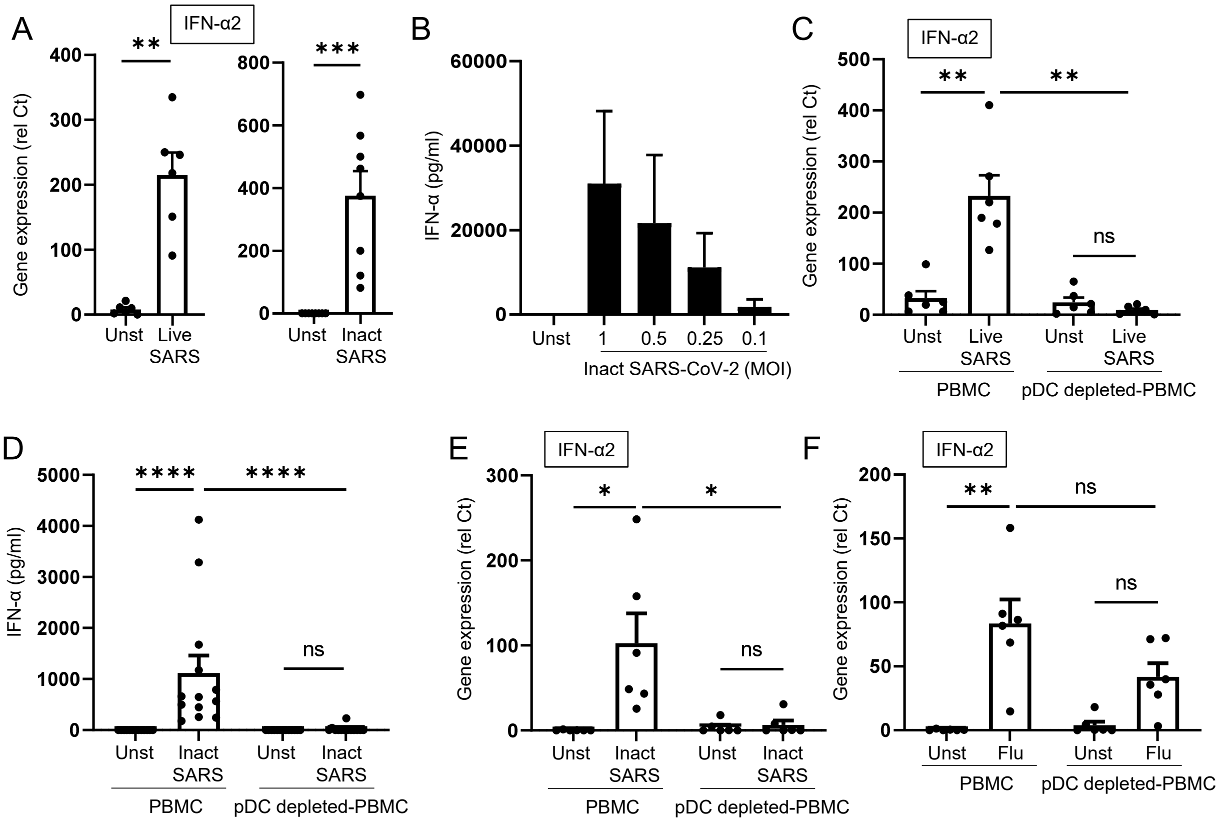

Supplementary figure 6: pDCs are the dominant producers of IFN-I in response to SARS-CoV-2 compared to Flu infection.

(**A**) pDCs purified from PBMCs of healthy donors (HDs)s (n=6 and 8) were cultured for 24 hours alone (unstimulated, unst) or with either live SARS-CoV-2 or inactivated (inact) SARS-CoV-2 at MOI 0.25. Gene expression of IFN-α was quantified by qPCR. (**B**) pDCs purified from PBMCs of HDs (n=3) were cultured for 24 hours alone (unst) with inactSARS-CoV-2 at MOI 1, 0.5, 0.25 and 0.1. IFN-α production was quantified by ELISA. (**C-F**) Total PBMCs or pDC-depleted PBMCs from HDs (n=6) were cultured for 24 hours alone (unst) or with either live SARS-CoV-2 at a MOI of 0.25, inact SARS-Cov_2 at a MOI of 0.25 or with inactivated Flu at a MOI of 2.. Gene expression and production of IFN-α were quantified by qPCR and ELISA respectively. All results are represented as means ± SEM. Statistical significance was evaluated using a Friedman test with Dunn's multiple comparisons posttest or a Mann-Whitney test *P < 0.05, **P < 0.01, ***P < 0.001. ns=not significant.

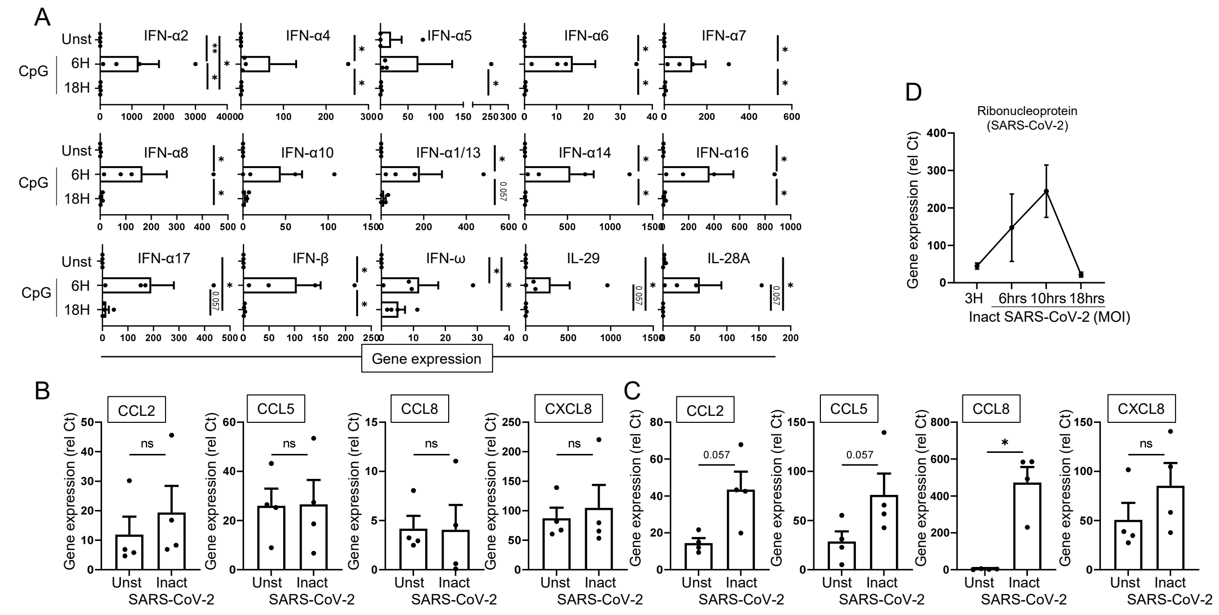

Supplementary figure 7: SARS-CoV-2 induces chemokines production by infected pDCs.

(**A**) pDCs purified from PBMCs of HDs (n=4) were incubated for 6h and 18h alone (unstimulated, unst) or with CpG C274 (0.5 μM). Expression levels of type I and type III IFNs were quantified by qPCR (rel Ct). (**B,C**) pDCs purified from PBMCs of HDs (n=4) were incubated for 6h (h) and 18h (i) alone (unst) or with inactivated (inact) SARS-CoV-2 (MOI 0.25). Expression levels of CCL2, CCL5, CCL8 and CXCL8 were quantified by qPCR (rel Ct). (**D**) pDCs purified from PBMCs of HD (n=4) were incubated for 3, 6, 10 or 18hours with inact SARS-CoV-2 at a MOI of 0.25. Ribonucleoprotein expression levels was quantified by qPCR.. All results are represented as means ± SEM. Statistical significance was evaluated using a Friedman test with Dunn's multiple comparisons posttest (d-f) or Mann-Whitney test (g). *P < 0.05, **P < 0.01, ***P < 0.001. ns=not significant.

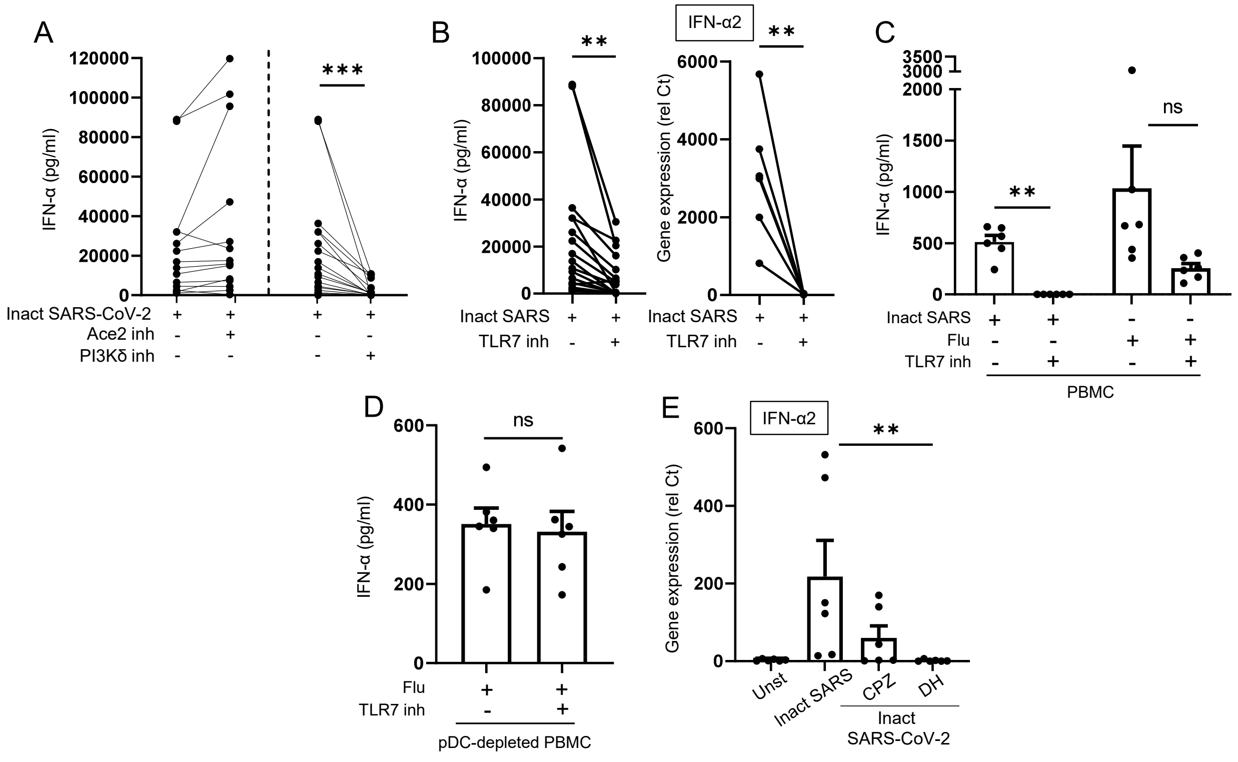

Supplementary figure 8: TLR7 sensing of SARS-CoV-2 by pDCs is necessary for the optimal induction of IFN-α by PBMC, while Flu can use other pathways

(**A-B**) pDCs purified from PBMCs of HDs (n=6 and 18) were cultured for 24 hours with t inactivated SARS-CoV-2 at a MOI of 0.25 either alone or in the presence of an ACE2 inhibitor (2 µM), a PI3Kδ inhibitor CAL-101 (10 µM) or a TLR7 inhibitor IRS661 (2 µM). Production and gene expression of IFN-α were quantified by ELISA and qPCR respectively. (**C**) PBMC were incubated with either inactivated (inact) SARS-CoV-2 (MOI=0.25) or Flu (MOI=2) in the presence or not of a TLR7 inhibitor IRS661 (2 µM). Production of IFN-α were quantified by ELISA. (**D**) pDC-depleted PBMC were incubated with Flu (MOI=2) alone or in the presence of a TLR7 inhibitor IRS661 (2 µM). IFN-α production was quantified by ELISA. (**E**) pDCs purified from PBMCs of HDs (n=4 to 8) were cultured for 24 hours with inactivated SARS-CoV-2 either alone or in the presence of chlathrin inhibitor chlorpromazine (CPZ, 30 µM) or dynamin inhibitor dynasore hydrate (DH, 100 µM). Gene expression of IFN-α2 was quantified by qPCR. All results are represented as means ± SEM. Statistical significance was evaluated using a Friedman test with Dunn's multiple comparisons posttest or a Mann-Whitney test *P < 0.05, **P < 0.01, ***P < 0.001. ns=not significant.

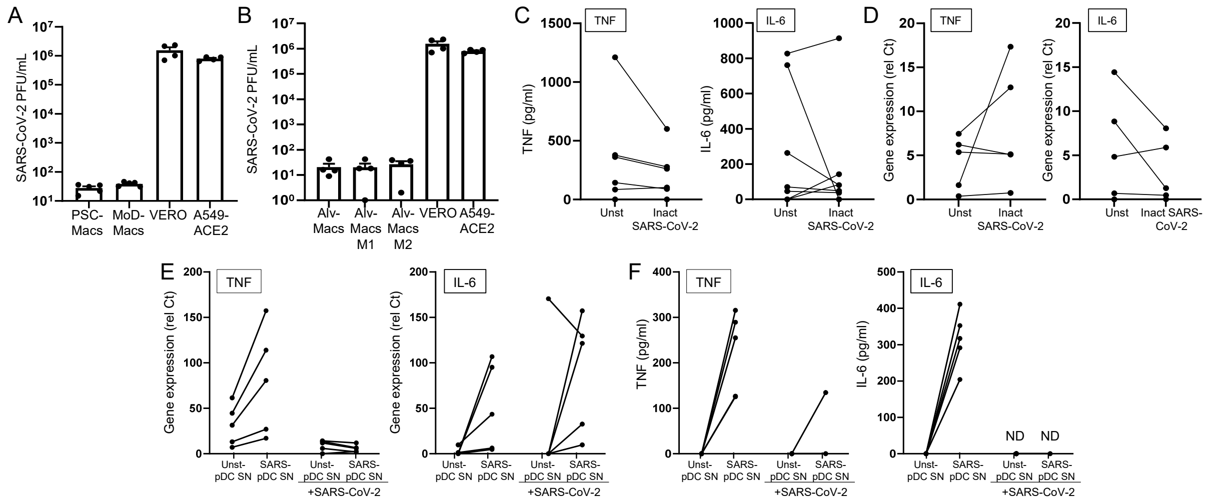

Supplementary figure 9: SARS-CoV-2 minimally activate macrophage response.

(**A**) Pluripotent stem cells (PSCs) derived macrophages (PSC-Macs), monocyte derived macrophages (MoD-Macs), VERO cells and A549-ACE2 cells were infected for 48h with live SARS-CoV-2 at a MOI of 0.1. (**B**) Alveolar macrophages isolated from primary human lung tissue (Alveo-Macs) were polarized or not in either M1 or M2 conditions (using LPS/IFN-γ or IL-4/IL-10, respectively) and infected for 48h with live SARS-CoV-2 at 0.1 MOI. (**C,D**) Macrophages purified from PBMCs of HDs (n=8 and 5) were cultured alone (unst) or withinactivated (inact) SARS-CoV-2 for 24 hours. Production and gene expression of TNF and IL-6 were quantified by ELISA and qPCR respectively. (**E,F**) Macrophages purified from PBMCs of HDs (n=5) were cultured for 24 hours with supernatants from either unstimulated pDCs (Unst-pDC SN) or from inactivated SARS-CoV-2-stimulated pDCs (SARS-pDC SN), followed by the addition of inactivated SARS-CoV-2 for 6 hours. Expression levels and production of TNF and IL-6 were quantified by qPCR and ELISA respectively. All results are represented as means ± SEM.

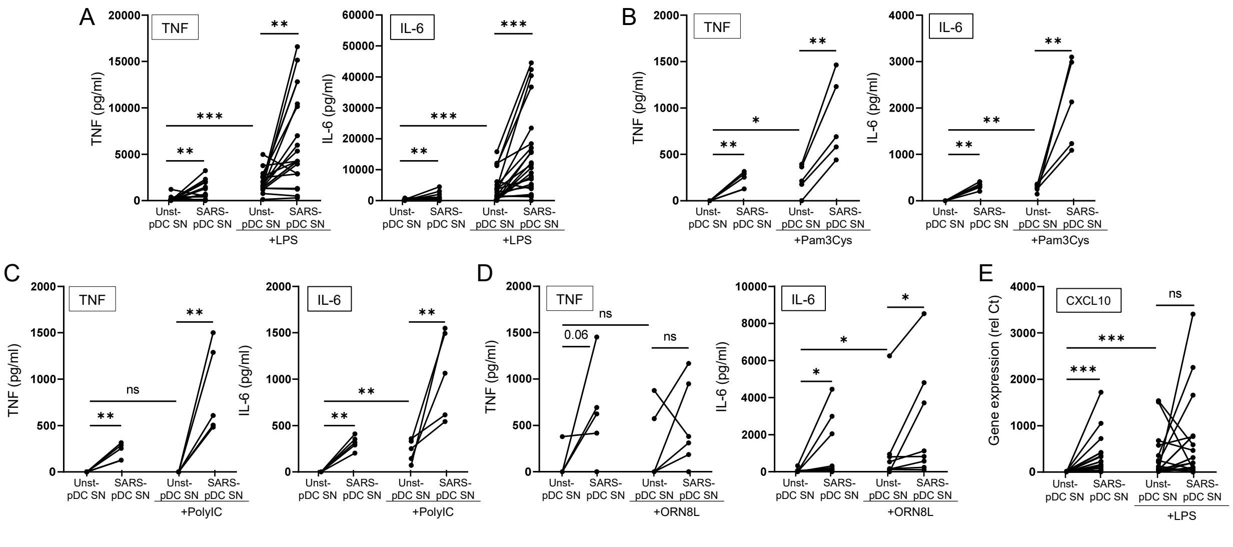

Supplementary figure 10: SARS-pDCs exacerbate macrophage activation.

(**A-D**) Macrophages purified from PBMCs of HDs (n=5 or 20) were cultured for 24 hours with the supernatant (SN) from either unstimulated pDCs (Unst-pDC SN) or from inactivated SARS-CoV-2-stimulated pDCs (SARS-pDC SN), followed by the addition of TLR agonists (LPS, Pam3Cys, PolyIC or ORN8L) for 6 hours. Production of TNF and IL-6 were quantified by ELISA. (**E**) Macrophages purified from PBMCs of HDs (n=20) were cultured for 24 hours with the supernatant (SN) from either unstimulated pDCs (Unst-pDC SN) or from inactivated SARS-CoV-2-stimulated pDCs (SARS-pDC SN), followed by the addition of LPS for 6 hours. Gene expression of CXCL10 was quantified by q-PCR. All results are represented as means ± SEM. Statistical significance was evaluated using a Mann-Whitney test. *P < 0.05, **P < 0.01, ***P < 0.001. ns=not significant.

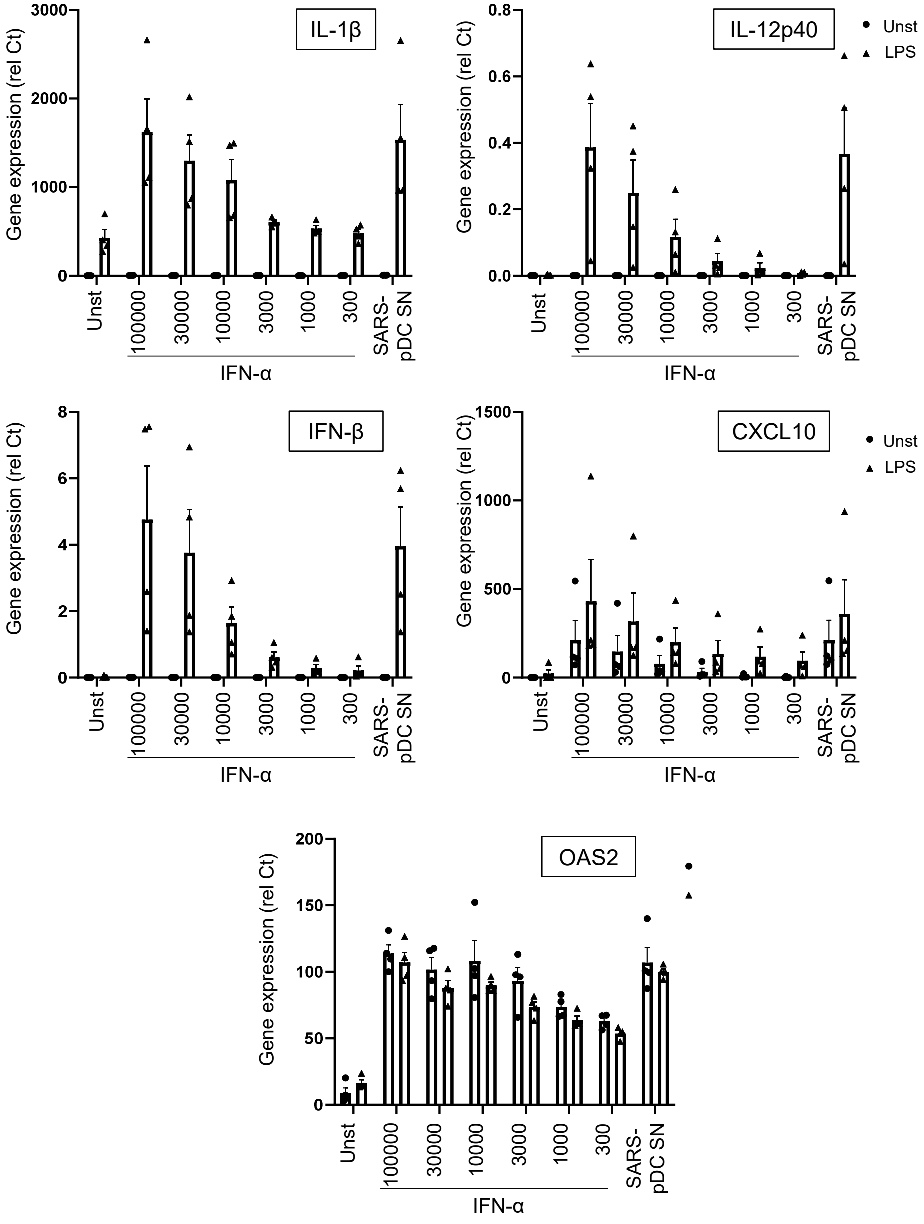

Supplementary figure 11: IFN-α induces inflammatory genes in dose-dependent manner.

Macrophages purified from PBMCs of HDs (n=4) were pre-incubated alone (unst) or with either different concentrations of IFN-α as indicated (pg/ml) or with supernatant of inactivated SARS-CoV-2- stimulated pDC (SARS-pDC SN), followed by the addition of LPS for 6hours.Gene expression levels of IL-1β, IL-12p40, IFN-β and CXCL10 were quantified in unstimulated macrophages (Unst.) and in macrophages incubated with either IFN-α (100000, 30000, 10000, 3000, 1000 and 300 pg/ml) or supernatants of SARS-pDCs (SARS-pDC SN) for 24 hours. When indicated, LPS was added for 3 hours (2 ng/ml). All results are represented as means ± SEM from HDs (n=4).

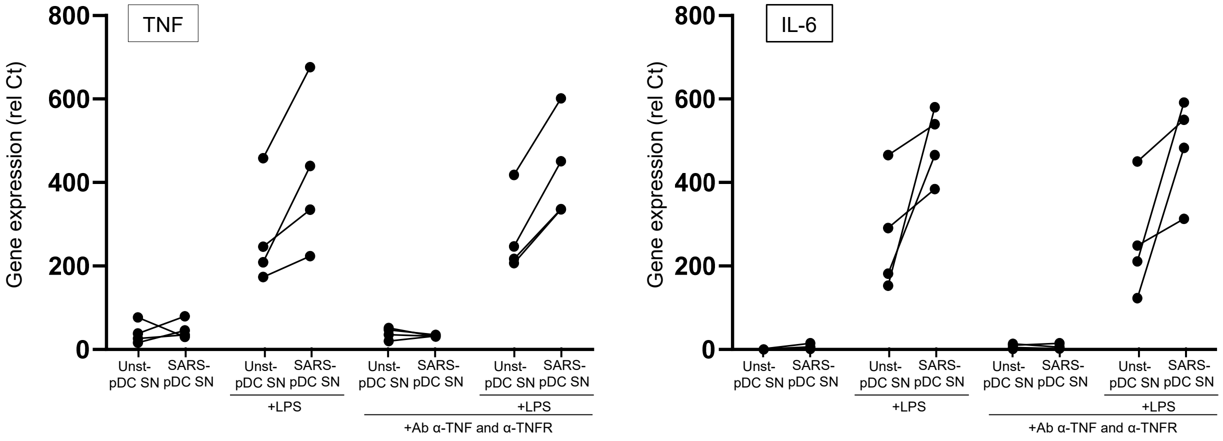

Supplementary figure 12: TNF produced by SARS-pDCs doesn't contribute to exacerbation of the macrophage response to LPS.

Macrophages purified from PBMCs of HDs (n=4) were cultured for 24 hours with the supernatant (SN) from either unstimulated pDCs (Unst-pDC SN) or from inactivated SARS-CoV-2-stimulated pDCs (SARS-pDC SN) in the presence of anti-TNFR antibody (10 µg/ml) and anti-TNF antibody (10 µg/ml), followed by the addition of TLR agonists for 6 hours. Expression levels of TNF and IL-6 quantified by qPCR. All results are represented as means ± SEM.

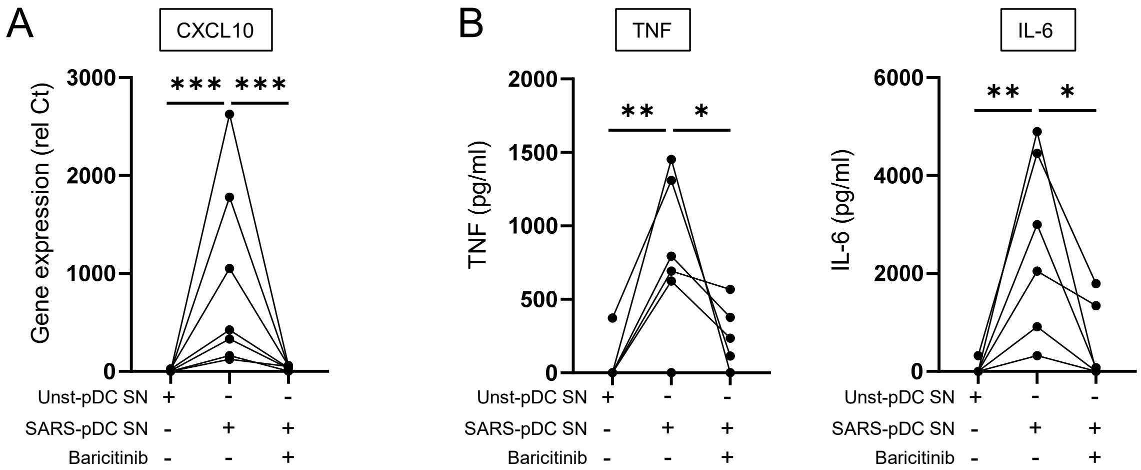

Supplementary figure 13: The activation of macrophage by supernatants of SARS-pDCs is blocked by baricitinib.

(**A,B**) Macrophages purified from PBMCs of HDs (n=7) were cultured for 24 hours with the supernatant (SN) from either unstimulated pDCs (Unst-pDC SN) or from inactivated SARS-CoV-2-stimulated pDCs (SARS-pDC SN) in the presence of baricitinib (2 μM). (**A**) Gene expression of CXCL10 was quantified by qPCR and (**B**) production of TNF and IL-6 were quantified by ELISA. All results are represented as means ± SEM. Statistical significance was evaluated using a Mann-Whitney test. *P < 0.05, **P < 0.01, ***P < 0.001.

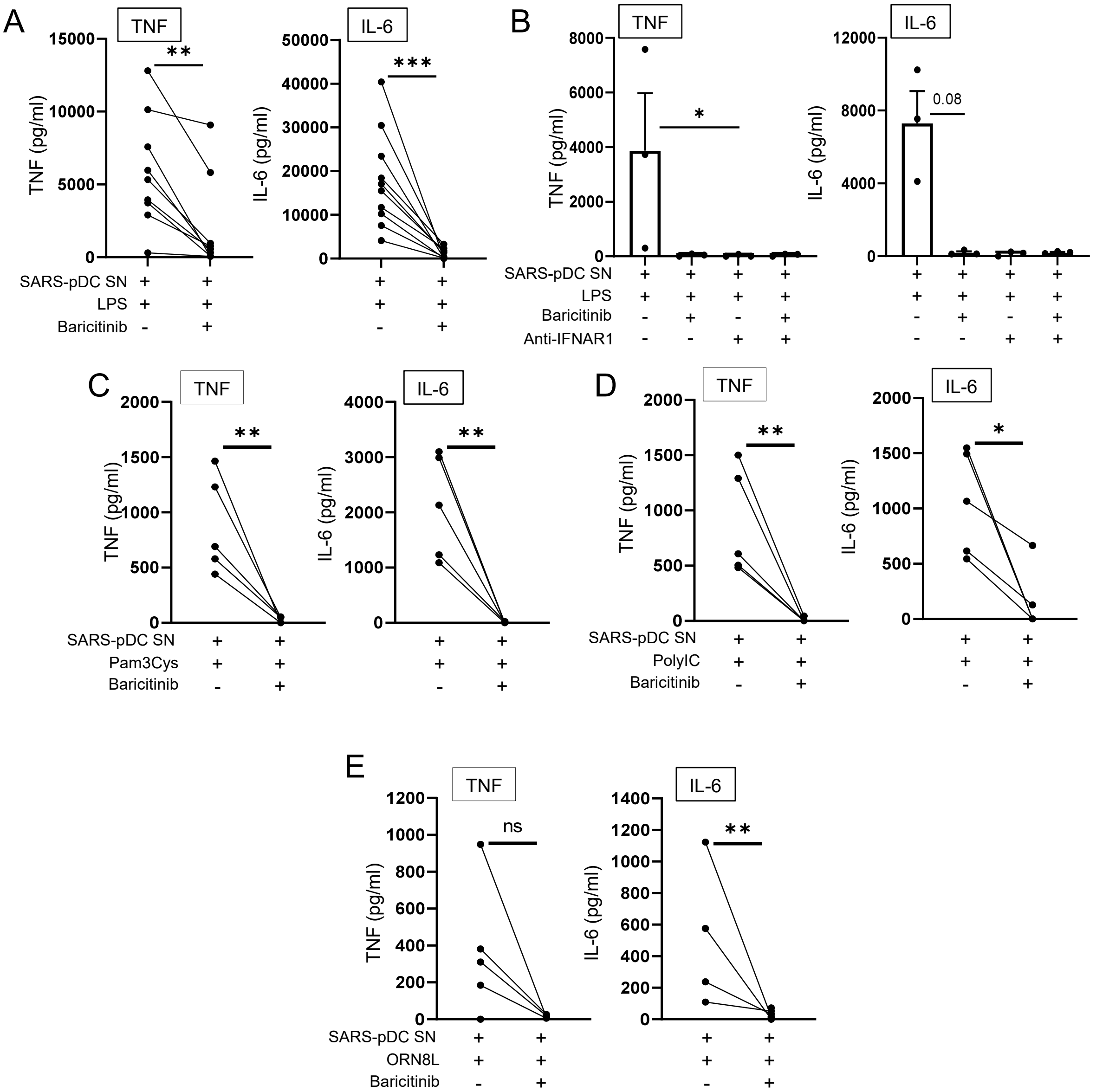

Supplementary figure 14: The induction of macrophage activation by supernatants of SARS-pDCs is blocked by baricitinib and requires IFN-I signaling.

(**A,B**) Macrophages purified from PBMCs of HDs (n=5-9) were pre-incubated for 24 hours with the supernatant of SARS-pDCs in the presence of baricitinib (2 µM) and/or anti-IFNAR antibody (2 µg/ml) followed by the addition of LPS for 6 hours. Production of TNF and IL-6 were quantified by ELISA. (**C-E**) Macrophages purified from PBMCs of HDs (n=5) were pre-incubated for 24 hours with the supernatant of SARS-pDCs in the presence of baricitinib (2 µM) followed by the addition of (**C**) Pam3Cys, (**D**) PolyIC or (**E**) ORN8L for 6 hours. Production of TNF and IL6 were quantified by ELISA. All results are represented as means ± SEM. Statistical significance was evaluated using a Mann-Whitney test (a-g) or Friedman test with Dunn's multiple comparisons posttest (h). $*P < 0.05$, $**P < 0.01$, $***P < 0.001$.

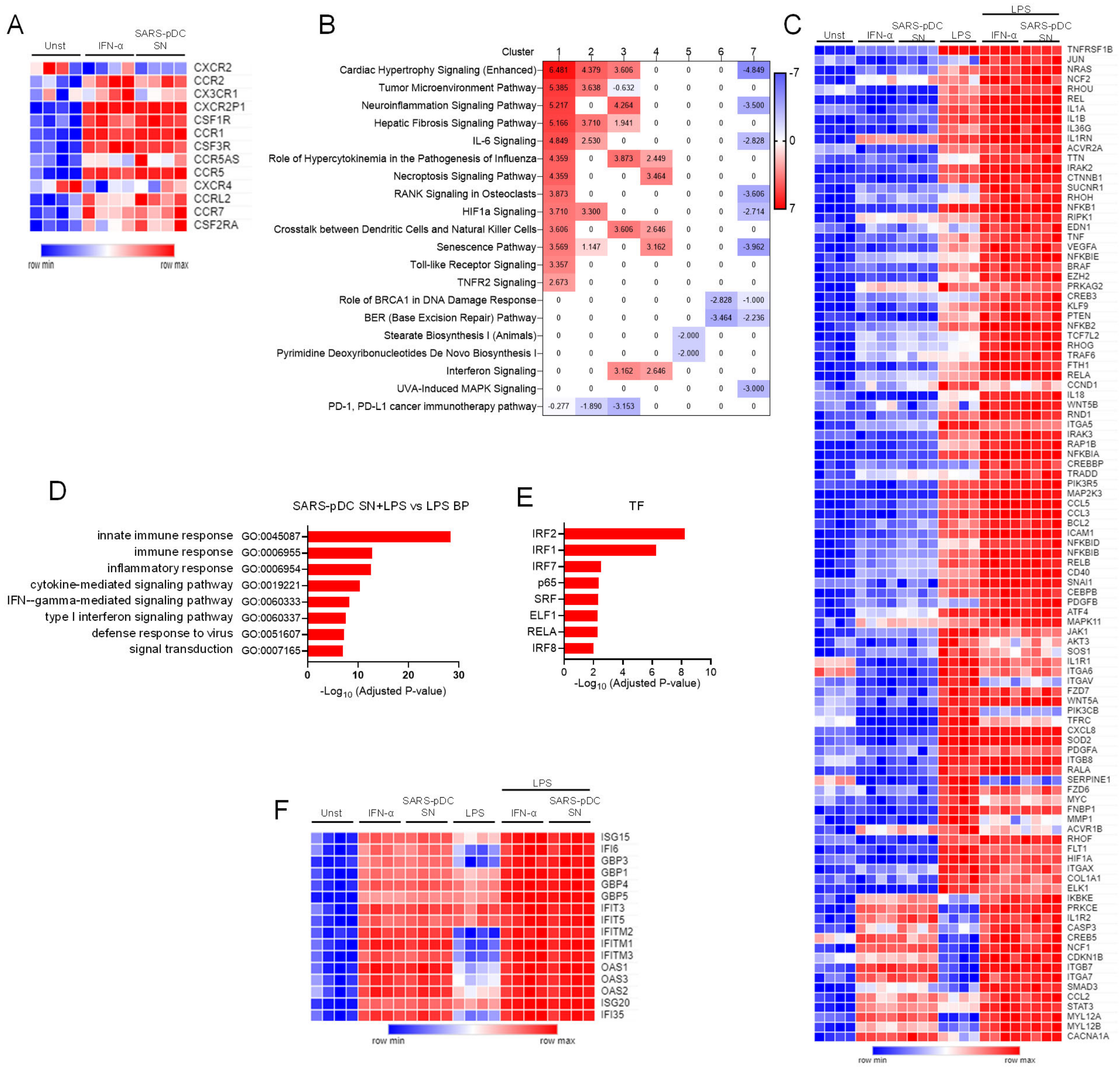

Supplementary figure 15: IFN-α increases TLR4-induced gene expression but not TLR4-mediated signaling pathway activation.

(**A**) Macrophages purified from PBMCs of HDs (n=3) were cultured alone (unst) or with either IFN-α or supernatant of inactivated SARS-CoV-2-stimulated pDCs (SARS-pDC SN) for 24 hours. Heat-map showing the expression of chemokine receptors in each condition.. (**B**) The top pathways analysis by IPA of the differentiated genes in each cluster from Fig. 4b. (**C**) Heat-map showing the fibrosis pathway associated gene expression in macrophages incubated in the indicated conditions. (**D,E**) The top GO_BP (D) and TF (E) enrichment of the differentiated expressed genes in the indicated conditions by InnateDB37. (**F**) Heat-map showing ISGs expression in macrophages in each indicated condition.

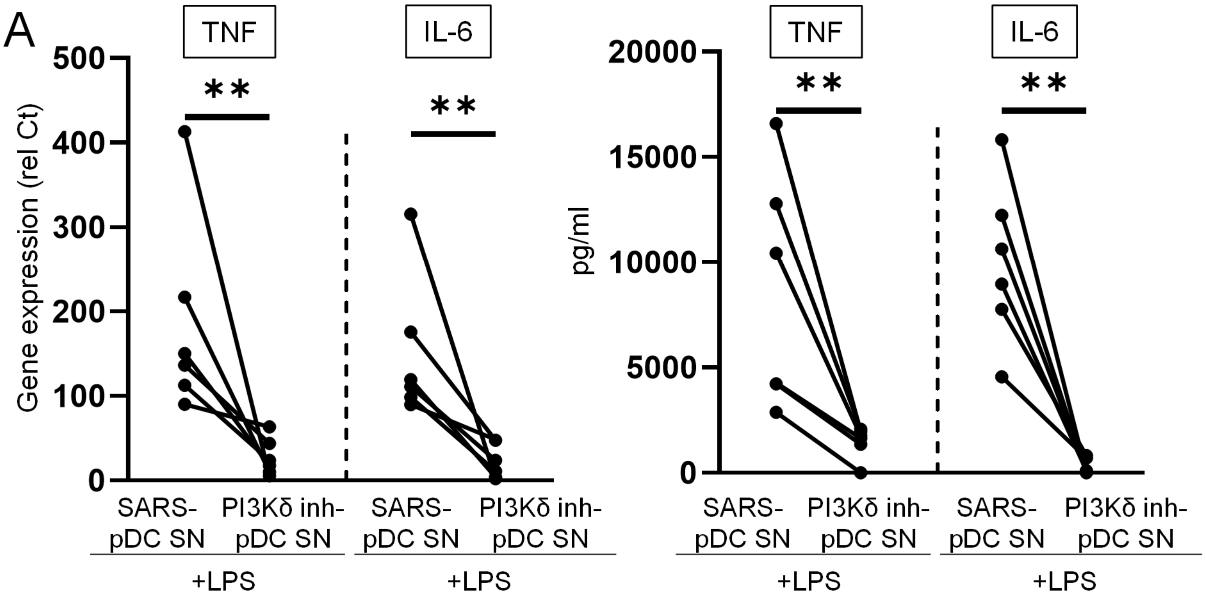

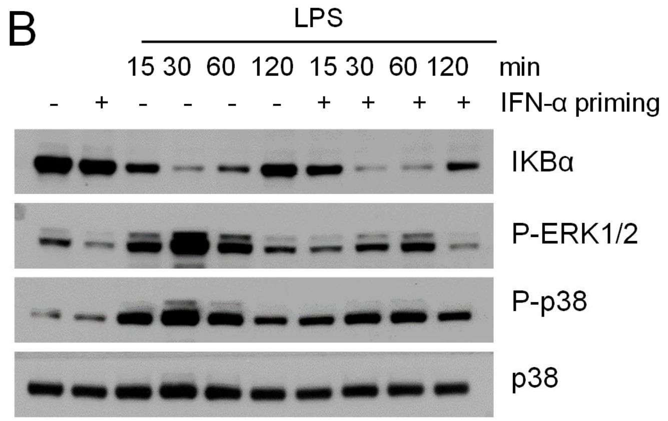

Supplementary figure 16: pDCs require TLR7 signaling and PI3Kδ in response to SARS-CoV-2 in order to superinduce macrophage response to stimuli, while IFN-α does not increase TLR4-mediated signaling pathway activation.

(**A**) Macrophages purified from PBMCs of HDs (n=6) were cultured for 24h with the supernatant of inactivated SARS-CoV-2-stimulated pDCs (SARS-pDC SN)alone or in the presence of PI3Kδ inhibitor CAL-101 (10 μM), followed by the addition of LPS for 6h. Gene expression levels and production of TNF and IL-6 were quantified by qPCR and ELISA respectively. (**B**) Immunoblot of NF-κB and MAPKs signaling in macrophages with whole cell lysates of the indicated conditions. All results are represented as means ± SEM. Statistical significance was evaluated using a Mann-Whitney test. *P < 0.05, **P < 0.01, ***P < 0.001

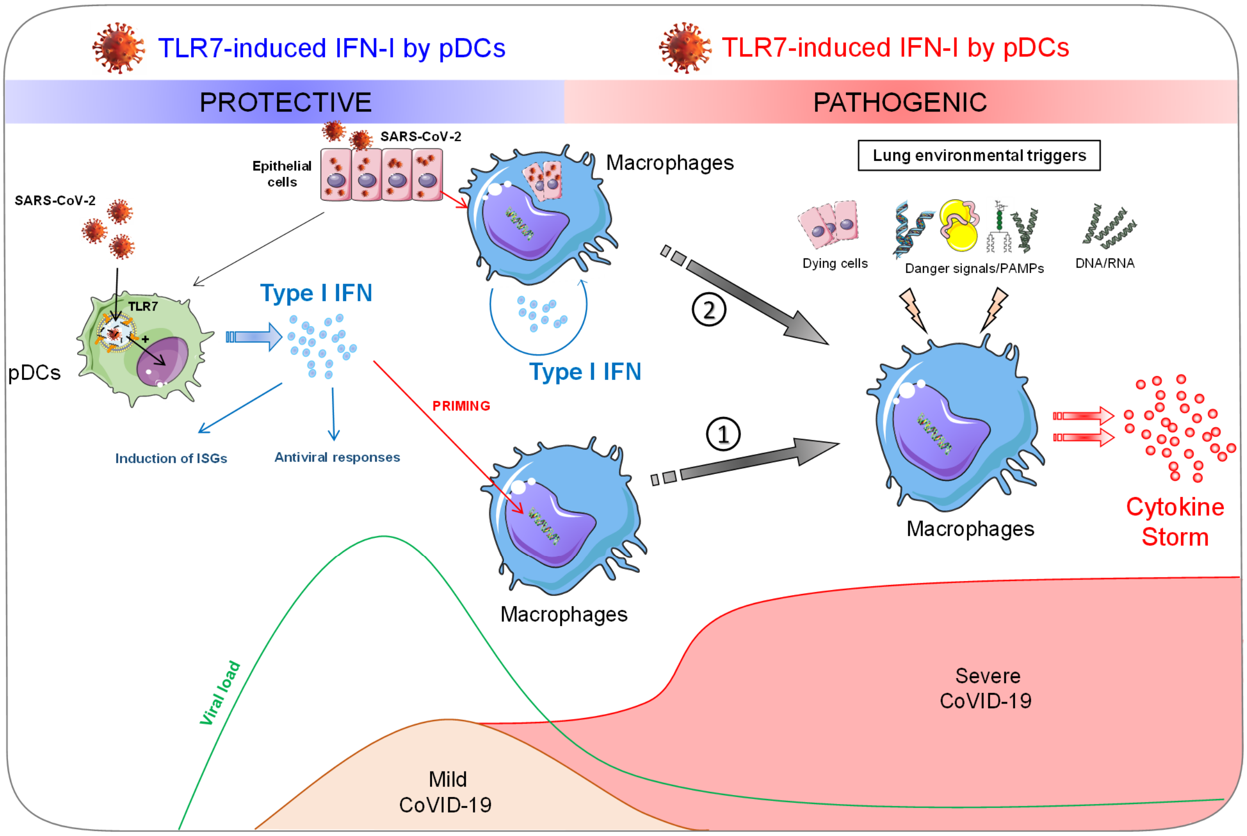

Supplementary figure 17: Sensing of SARS-CoV-2 and IFN-I production by pDCs promotes the cytokine storm by macrophages.

The proposed model is that the sensing of SARS-CoV-2 by TLR7 in pDCs is critical to develop an adequate anti-viral response in infected individual. However, in a subset of patients, lung infiltrating pDCs, also activated via TLR7, secrete IFN-I which then prime monocyte-derived macrophages which under the stimulation of environmental factors, produce large quantities of cytokines with deleterious consequences for the patients. A lack of proper pDC response can also favor the infection of epithelial cells which in turn can activate the macrophages to produce IFN-I and/or inflammatory cytokines.
